# Identification of Chemical Constituents in *Blumea balsamifera* Using UPLC–Q–Orbitrap HRMS and Evaluation of Their Antioxidant Activities

**DOI:** 10.3390/molecules28114504

**Published:** 2023-06-01

**Authors:** Liping Dai, Shengnan Cai, Dake Chu, Rui Pang, Jianhao Deng, Xilong Zheng, Wei Dai

**Affiliations:** 1 College of Traditional Chinese Medicine Resources, Guangdong Pharmaceutical University, Yunfu 527325, China; 2 Experimental Center of Yunfu Campus, Guangdong Pharmaceutical University, Yunfu 527325, China

**Keywords:** *Blumea balsamifera*, UPLC–Q–Orbitrap HRMS, chemical constituents, mass spectrometry fragmentation patterns, antioxidant activities

## Abstract

*Blumea balsamifera* (L.) DC., a perennial herb in the Asteraceae family native to China and Southeast Asia, has a notable history of medicinal use due to its pharmacological properties. Using UPLC–Q–Orbitrap HRMS techniques, we systematically investigated the chemical constituents of this plant. A total of 31 constituents were identified, of which 14 were flavonoid compounds. Significantly, 18 of these compounds were identified in *B. balsamifera* for the first time. Furthermore, the mass spectrometry fragmentation patterns of significant chemical constituents identified in *B. balsamifera* were analyzed, providing important insights into their structural characteristics. The in vitro antioxidative potential of the methanol extract of *B. balsamifera* was assessed using DPPH and ABTS free-radical-scavenging assays, total antioxidative capacity, and reducing power. The antioxidative activity exhibited a direct correlation with the mass concentration of the extract, with IC_50_ values of 105.1 ± 0.503 μg/mL and 12.49 ± 0.341 μg/mL for DPPH and ABTS, respectively. For total antioxidant capacity, the absorbance was 0.454 ± 0.009 at 400 μg/mL. In addition, the reducing power was 1.099 ± 0.03 at 2000 μg/mL. This study affirms that UPLC–Q–Orbitrap HRMS can effectively discern the chemical constituents in *B. balsamifera*, primarily its flavonoid compounds, and substantiates its antioxidative properties. This underscores its potential utility as a natural antioxidant in the food, pharmaceutical, and cosmetics sectors. This research provides a valuable theoretical basis and reference value for the comprehensive development and utilization of *B. balsamifera* and expands our understanding of this medicinally valuable plant.

## 1. Introduction

Oxidative stress, a pathological condition induced by an imbalance between reactive oxygen species’ (ROS) production and the biological system’s ability to detoxify these reactive intermediates, contributes to several chronic diseases such as cancer, diabetes, cardiovascular diseases, neurodegenerative disorders, and aging. Antioxidants have the potential to quell this imbalance by neutralizing ROS, thereby preventing or delaying cellular damage [1]. Plant–derived antioxidants, owing to their availability, safety, and therapeutic efficacy, have been the focus of many studies. Plants, with their extensive repertoire of phytochemicals such as polyphenols, flavonoids, and terpenoids, among others, have demonstrated significant antioxidant activity [2,3,4].

*Blumea balsamifera* (L.) DC., a herbaceous plant from the Asteraceae family, is extensively distributed in tropical and subtropical regions, including countries such as China, Thailand, Vietnam, the Philippines, and Malaysia [5]. The first documented use of it as a medicine in China can be traced back to 652 A.D., primarily for the treatment of conditions such as colds, wind–heat headache, rheumatic arthralgia, irregular menstruation, and dysmenorrhea [6,7,8]. The plant is primarily composed of volatile oils, flavonoids, terpenes, phenylpropanoids, and sterols, with volatile oils, flavonoids, and sesquiterpenes being its main chemical constituents. These components have been linked to the plant’s pharmacological effects, including antibacterial, anti–inflammatory, anticancer, burn treatment, neuroinflammation suppression, hypoglycemic, antioxidant, anti–obesity, and antiviral activities [9,10,11,12,13,14]. However, the material basis and action mechanisms behind these efficacies remain to be fully elucidated, highlighting the need for more comprehensive research.

The chemical constituents in herbal medicines are the material basis for their efficacy and the key indicators for herbal medicines’ quality control. The complexity of herbal medicines’ chemical constituents poses challenges for their rapid identification and determination. Ultra-high-performance liquid chromatography–quadrupole/orbitrap high–resolution mass spectrometry (UPLC–Q–Orbitrap HRMS) has been widely used for the qualitative and quantitative analysis and quality control of herbal medicines [15,16,17,18]. The technique offers higher efficiency and reliability compared to traditional methods of separation and identification of herbal medicine’s chemical constituents. For *B. balsamifera*, the use of UPLC–Q–Orbitrap HRMS would allow us to identify components that might not be detected using conventional methods, providing a more comprehensive understanding of the plant’s chemical composition and potential bioactivities.

In this study, we employed UPLC–Q–Orbitrap–MS to perform a comprehensive scan of the methanol extract of *B. balsamifera* in both positive and negative ion modes. Using Xcalibur 4.1 software, we obtained chromatographic information, analyzed it based on full–scan MS and MS^2^ information, and matched it with databases (mzVault and mzCloud). We identified the constituents by comparing the fragmentation patterns and literature reports, as well as by comparing them with reference standards. Furthermore, we evaluated the antioxidative activity of the methanol extract of *B. balsamifera* by assessing its DPPH and ABTS free radical scavenging abilities, total antioxidant capacity, and total reducing power. The obtained information on the chemical constituents and antioxidative activity of *B. balsamifera* will provide new insights for the quality control and clinical applications of this medicinal plant.

## 2. Results

### 2.1. Identification of Chemical Constituents by UPLC–Q–Orbitrap HRMS

The analysis using UPLC-Q-Orbitrap HRMS resulted in identifying a total of 31 compounds in the extract of *B. balsamifera*. These compounds comprise 14 flavonoids, 5 organic acids, 3 quinones, 2 alkaloids, and 7 other compounds. Table 1 provides detailed information regarding these compounds. The flavonoids, accounting for 45% of the total identified compounds, suggest that they are the main chemical constituents in *B. balsamifera*. The identified flavonoids include hemiphloin, taxifolin, rutin, quercetin–3β–D–glucoside, quercetin, quercetin–3–arabinoside, cynaroside, isorhamnetin–3–O–glucoside, eriodictyol, padmatin, isorhamnetin, naringenin, 7–O–methylaromadendrin, and sakuranetin. The identified organic acids include caffeic acid, tuberonic acid glucoside, 1,3–dicaffeoylquinic acid, chlorogenic acid, and 4–oxo–5–phenylpentanoic acid. The identified quinones are aurantio–obtusin, cryptotanshinone, and tanshinone IIA. The identified alkaloids are Aurantiamide acetate and N–phenyl–1–naphthylamine. Other compounds identified include α,α–trehalose, D–(−)–fructose, gentisic acid 5–O–β–glucoside, fraxetin, hematoxylin, nootkatone, and citroflex A–4. Notably, a total of 18 compounds were successfully identified in *B. balsamifera*, representing the first report of their presence in this plant species. This discovery provides valuable insights and contributes to the expanding chemical knowledge of *B. balsamifera*.

### 2.2. Identification and Fragmentation Patterns of Major Chemical Constituents

#### 2.2.1. Identification and Fragmentation Patterns of Flavonoids

**Figure 1:**
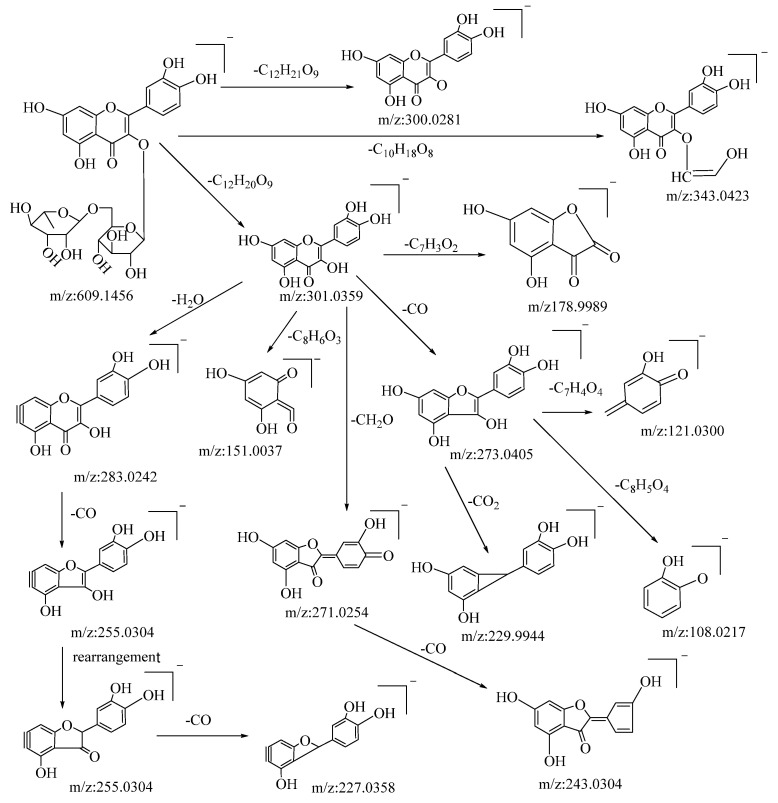
Possible fragmentation pathways of rutin in negative ion mode.

Flavonoids are among the primary active compounds found in *B. balsamifera*, with 14 flavonoid compounds being identified in both positive and negative ion modes. The fragmentation of flavonoids through mass spectrometry is generally characterized by the cleavage of flavonoid glycosides into glycosides and flavonoid aglycone fragments. Further fragmentation of flavonoid aglycones can be categorized into two primary classes: the Retro–Diels–Alder (RDA) cleavage of the ring, resulting in the division of the flavonoid aglycones into two distinct fragments, and the elimination of certain smaller molecular fragments, including CO, CHO, C_3_O_2_, CO_2_, and C_2_HO, among others [48]. The specific fragmentation pathways and resultant products are influenced by several factors, such as the reaction conditions, molecular structure, and the underlying reaction mechanism. Rutin is a type of flavonoid glycoside. In negative ion mode, its quasi-molecular ion peak is at *m*/*z*: 609.1456 [M−H]^−^, with its molecular formula deduced as C_27_H_30_O_16_ using Xcalibar 4.0 software. Upon induced collision dissociation, it loses to form three kinds of fragment ions. Firstly, it loses a sugar moiety C_12_H_21_O_9_ to form a fragment *m*/*z*: 300.0281. Secondly, it undergoes a cleavage from the sugar moiety, losing C_10_H_18_O_8_ to form a fragment *m*/*z*: 343.0423. Thirdly, it loses a sugar moiety C_12_H_20_O_9_ to form a fragment ion *m*/*z*: 301.0359, which, through five types of cleavage pathways, yields different fragments. These pathways include: losing a molecule C_7_H_3_O_2_ at the C–ring’s C–2 site to produce fragment *m*/*z*: 178.9989; losing a water molecule on the A–ring to yield fragment *m*/*z*: 283.0242, which continues to cleave a molecule of CO from the C–ring to yield fragment *m*/*z*: 255.0304 and, through rearrangement, further loses a molecule of CO from the C–ring to yield fragment *m*/*z*: 227.0358; undergoing a RDA cleavage on the C–ring, losing C_8_H_6_O_3_, to yield fragment *m*/*z*: 151.0037; losing a molecule of CH_2_O on the C–ring to yield fragment *m*/*z*: 271.0254, which continues to lose a molecule of CO on the C–ring to yield fragment *m*/*z*: 243.0307; and cleaving a CO molecule at the carbonyl on the C–ring to yield fragment *m*/*z*: 273.0405, which continues to cleave through three pathways, losing C_7_H_4_O_4_, CO_2_, and C_8_H_5_O_4_, respectively, to yield fragments *m*/*z*: 121.0300, *m*/*z*: 229.9944, and *m*/*z*: 108.0217. By comparison with the corresponding literature references [49], and based on mass spectral data analysis and comparison with reference substances, it is speculated that this compound is rutin. The possible mass spectral cleavage pathways are shown in Figure 1.

**Figure 2:**
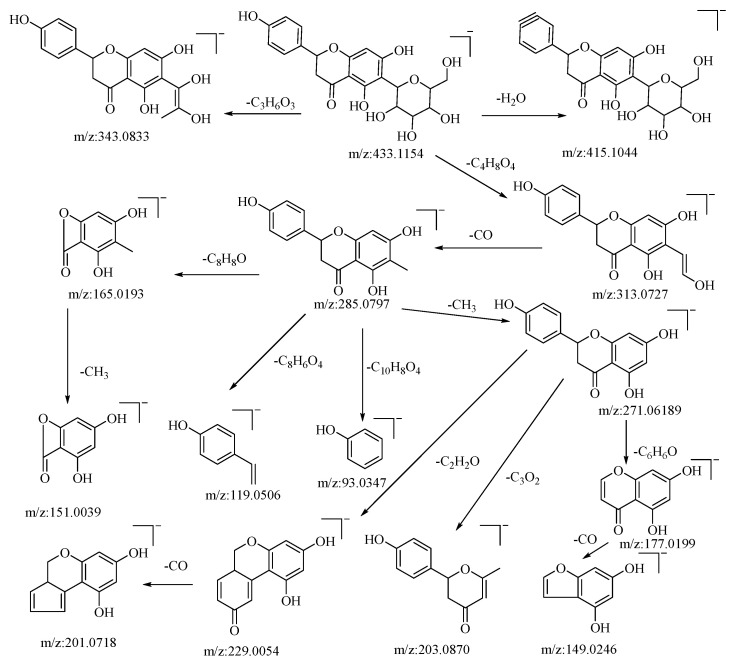
Possible mass spectrometry fragmentation pathway of hesperidin in negative ion mode.

Hesperidin is a flavonoid glycoside. In negative ion mode, the quasi-molecular ion peak is at *m*/*z*: 433.1154 [M−H]^−^. Using Xcalibar 4.0 software, the molecular formula is deduced to be C_21_H_22_O_10_. Upon collision-induced dissociation, it loses C_3_H_6_O_3_ and C_4_H_8_O_4_ to yield secondary fragment ions *m*/*z*: 343.0833 and *m*/*z*: 313.0727, which are characteristic fragment ions of hexose flavonoid glycosides. The *m*/*z*: 313.0727 ion loses CO from the sugar moiety residue to yield fragment ion *m*/*z*: 285.0797, with four cleavage pathways. Firstly, it loses a methyl group from the C–ring to form fragment *m*/*z*: 271.0619, which continues to cleave in three pathways: losing the B–ring to yield fragment *m*/*z*: 177.0199, which further loses a carbonyl group from the A–ring to yield fragment *m*/*z*: 149.0246; cleaving C_3_O_2_ from the A–ring to yield fragment *m*/*z*: 203.0870; and losing C_2_H_2_O from the C–ring to yield fragment *m*/*z*: 229.0054, which further loses a molecule of CO from the B–ring to yield fragment *m*/*z*: 201.0718. Secondly, it loses the A–ring and C–ring from the connection between the A and B rings to yield fragment *m*/*z*: 93.0347. Thirdly, a RDA cleavage occurs on the C–ring, cleaving C_8_H_8_O from C–1 and C–3 to yield fragment *m*/*z*: 165.0196, which continues to lose a methyl group from the A–ring to yield fragment *m*/*z*: 151.0039. Fourthly, cleaving C_8_H_6_O_4_ from the C–ring yields fragment *m*/*z*: 119.0506. The pseudomolecular ion peak *m*/*z*: 433.1154 loses a water molecule from the B–ring to form fragment *m*/*z*: 415.1044. By comparison with the corresponding literature references [50,51], and based on mass spectral data analysis, this compound is hypothesized to be hesperidin. The possible mass spectral cleavage pathways are illustrated in Figure 2.

#### 2.2.2. Identification and Fragmentation Patterns of Organic Acids

**Figure 3:**
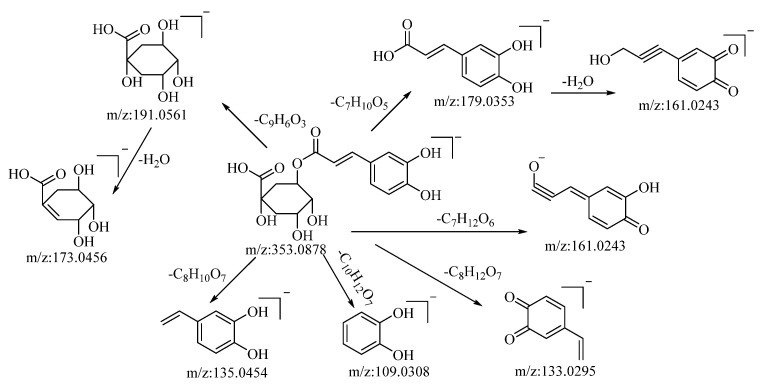
Possible mass fragmentation pathways of chlorogenic acid in negative ion mode.

Through both positive and negative ion modes, five organic acid compounds were successfully identified in *B. balsamifera*. Notably, chlorogenic acid demonstrated a quasi–molecular ion peak at *m*/*z*: 353.0878 [M−H]^−^ in the negative ion mode. The molecular formula C_16_H_18_O_9_ was discerned utilizing the Xcalibur 4.0 software. Upon collision-induced dissociation, the molecule generated a variety of fragment ions, as described below: fragmentation through ester bond breakage resulted in a fragment at *m*/*z*: 191.0561, which, upon further loss of a water molecule, yielded a fragment at *m*/*z*: 173.0456. Another fragmentation process involving ester bond cleavage produced a fragment at *m*/*z*: 179.0353, which, after the loss of a water molecule, formed a fragment at *m*/*z*: 161.0243. Cleavage to the left of the carbonyl group, resulting in the loss of C_7_H_12_O_6_, generated a fragment at *m*/*z*: 161.0243. The loss of C_8_H_12_O_7_ led to the formation of a fragment at *m*/*z*: 133.0295. Cleavage at the alkyl group of the phenyl ring, which resulted in the loss of C_10_H_12_O_7_, generated a fragment at *m*/*z*: 109.0308. Lastly, cleavage to the left of the carbon–carbon double bond, accompanied by the loss of C_8_H_10_O_7_, produced a fragment at *m*/*z*: 135.0454. Drawing upon mass spectral data analysis, in comparison with the relevant literature references [52], and in juxtaposition with reference standards, the compound in question is hypothesized to be chlorogenic acid. The potential mass spectral cleavage pathways are depicted in Figure 3.

#### 2.2.3. Identification and Fragmentation Pathways of Alkaloids

**Figure 4:**
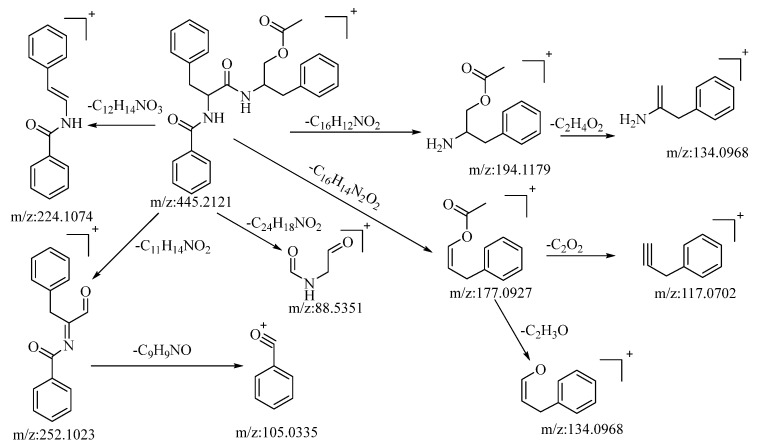
The possible mass spectrometry fragmentation pathway of aurantiamide acetate in positive ion mode.

The compound under consideration, aurantiamide acetate, exhibited a quasi-molecular ion peak at *m*/*z*: 445.2121 [M+H]^+^ in the positive ion mode. Its molecular formula, C_27_H_28_N_2_O_4_, was determined utilizing Xcalibur 4.0 software. Following collision-induced dissociation, it generated five distinct types of fragment ions as described below: the loss of C_16_H_12_NO_2_ yielded a fragment at *m*/*z*: 194.1179, which further underwent the loss of C_2_H_4_O_2_, resulting in a fragment at *m*/*z*: 134.0968. The loss of C_12_H_14_NO_3_ led to the formation of a fragment at *m*/*z*: 224.1074. The loss of C_11_H_14_NO_2_ produced a fragment at *m*/*z*: 252.1023. This fragment, upon further loss of C_9_H_9_NO, generated a fragment ion at *m*/*z*: 105.033, which was the most stable fragment ion peak under these conditions. The loss of C_24_H_18_NO_2_ resulted in a fragment at *m*/*z*: 88.5351. The loss of C_16_H_14_N_2_O_2_ yielded a fragment at *m*/*z*: 177.0927, which further lost C_2_O_2_ and C_2_H_3_O to yield fragments at *m*/*z*: 117.0702 and *m*/*z*: 134.0968, respectively. The loss of C_16_H_14_NO_2_ and C_2_H_3_O_2_ led to the formation of fragments at *m*/*z*: 194.1179 and *m*/*z*: 134.0968, respectively. Based on mass spectral data analysis and comparison with the related literature reference [53], this compound is conjectured to be aurantiamide acetate. The potential mass spectral cleavage pathways are illustrated in Figure 4.

#### 2.2.4. Identification and Fragmentation Pathways of Quinones

**Figure 5:**
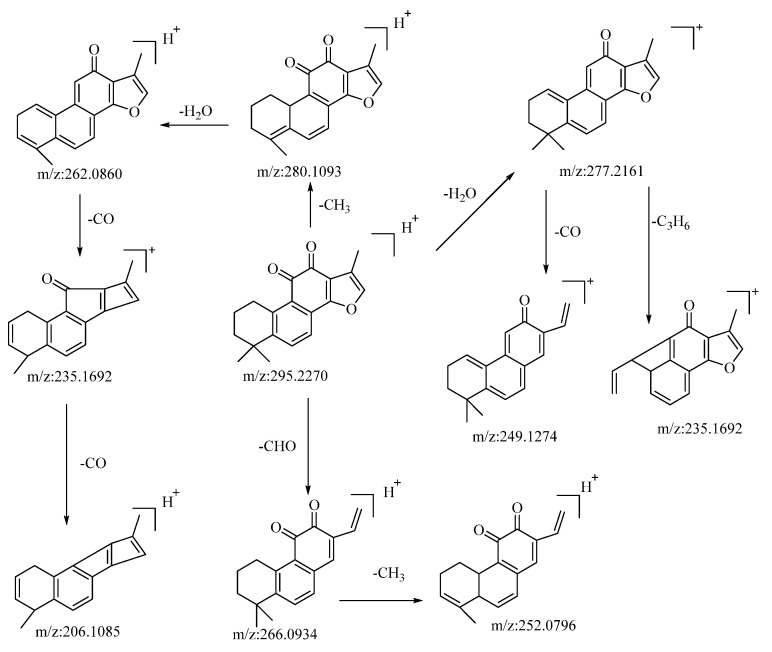
The possible mass fragmentation pathway of tanshinone IIA in positive ion mode.

The compound tanshinone IIA, operating in the positive ion mode, presented a quasi-molecular ion peak at *m*/*z*: 295.2207 [M−H]^−^. Its molecular formula, C_19_H_18_O_3_, was ascertained using the Xcalibur 4.0 software. Subsequent to collision-induced dissociation, it gave rise to three distinct types of fragment ions, as detailed below: the loss of a molecule of H_2_O resulted in a fragment at *m*/*z*: 277.2161. Further losses of CO and C_3_H_6_ yielded fragments at *m*/*z*: 249.1274 and *m*/*z*: 235.1692, respectively. Dimethyl cleavage involving the loss of a CH_3_ molecule produced a fragment at *m*/*z*: 280.1093. A subsequent loss of H_2_O gave rise to a fragment at *m*/*z*: 262.0860. Consecutive losses of a CO molecule resulted in fragments at *m*/*z*: 235.1692 and *m*/*z*: 206.1085, respectively. The loss of a molecule of CHO led to the formation of a fragment at *m*/*z*: 266.0934. A subsequent loss of CH_3_ generated a fragment at *m*/*z*: 252.0796. Drawing upon the analysis of mass spectral data and comparisons with the related literature references [54], the compound is proposed to be tanshinone IIA. The potential mass spectral cleavage pathways are illustrated in Figure 5.

### 2.3. Antioxidant Activity Determination

#### 2.3.1. DPPH Radical Scavenging Activity

**Figure 6:**
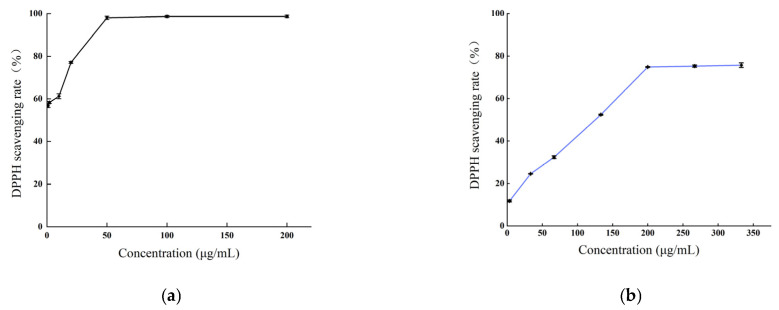
The DPPH radical scavenging activities of (**a**) the ascorbic acid control and (**b**) *B. balsamifera* methanol extract.

The DPPH radical scavenging capacity of the methanol extract of *B. balsamifera*, alongside an ascorbic acid control, is depicted in Figure 6. As illustrated, an increased concentration of the various samples corresponded with a gradual increase in DPPH radical scavenging activity, eventually reaching a plateau at a specific concentration. For ascorbic acid, the DPPH scavenging rate stabilized at a concentration of 50 μg/mL, reaching a peak value at 200 μg/mL with a maximum scavenging rate of 98.72%. The methanol extract exhibited a rapid increase in scavenging rate against DPPH radicals at concentrations ranging from 3.33 to 200 μg/mL. At 200 μg/mL, the scavenging rate tended to stabilize, and the maximum scavenging rate was 75.72% at 333 μg/mL. which was lower than that of ascorbic acid.

The value of the half-inhibitory concentration (IC_50_) of the sample was calculated. A smaller IC_50_ value indicated stronger antioxidant activity. The IC_50_ values for ascorbic acid and the methanol extract in scavenging DPPH radicals were as follows: ascorbic acid (0.98 ± 0.073 μg/mL) < methanol extract (105.1 ± 0.503 μg/mL), as shown in Table 2.

#### 2.3.2. ABTS Radical Scavenging Activity

**Figure 7:**
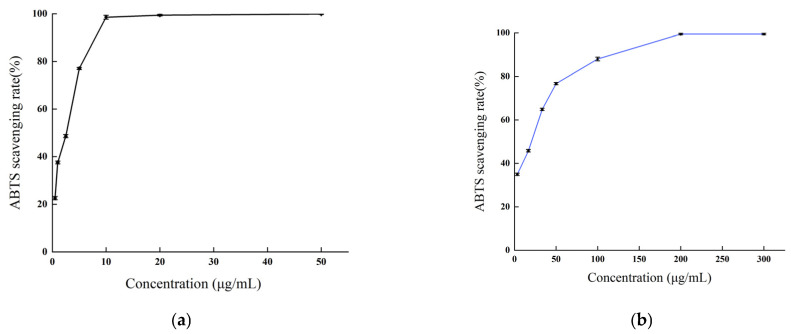
The ABTS radical scavenging activities of (**a**) the ascorbic acid control and (**b**) *B. balsamifera* methanol extract.

As depicted in Figure 7, the methanol extract of *B. balsamifera* demonstrated a potent scavenging activity against ABTS radicals. This scavenging capability exhibited a positive correlation with the sample concentration within a certain range. The scavenging rate of ascorbic acid against ABTS radicals reached a plateau at a concentration of 10 μg/mL and attained a maximum value at 50 μg/mL, with the peak scavenging rate being 99.87%. The methanol extract displayed a maximum scavenging rate exceeding 99% at a concentration of 200 μg/mL, a value comparable to that of ascorbic acid.

The IC_50_ values, which signify the potency of antioxidant activity of the ascorbic acid and methanol extract in scavenging ABTS radicals are as follows, listed from smallest to largest: ascorbic acid (1.774 ± 0.036 μg/mL) < methanol extract (12.49 ± 0.341 μg/mL), as indicated in Table 2.

#### 2.3.3. Total Antioxidant Capacity Determination

**Figure 8:**
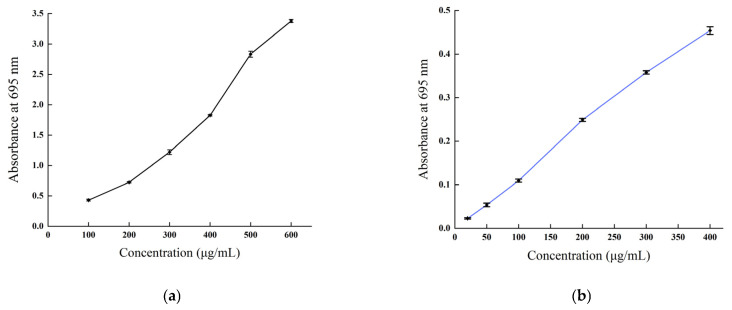
Total antioxidant capacity of (**a**) the ascorbic acid control and (**b**) *B. balsamifera* methanol extract.

The total antioxidant capacity of *B. balsamifera* methanol extract and ascorbic acid control are shown in Figure 8. As shown in the figure, the linear total antioxidant capacity of ascorbic acid and methanol extract exhibited an apparent dose–response relationship. The total antioxidant capacity of ascorbic acid reached the highest absorbance value of 3.38 ± 0.027 at a concentration of 600 µg/mL, while the methanol extract reached a maximum absorbance value of 0.454 ± 0.009 at 400 μg/mL.

#### 2.3.4. Reducing Power Determination

**Figure 9:**
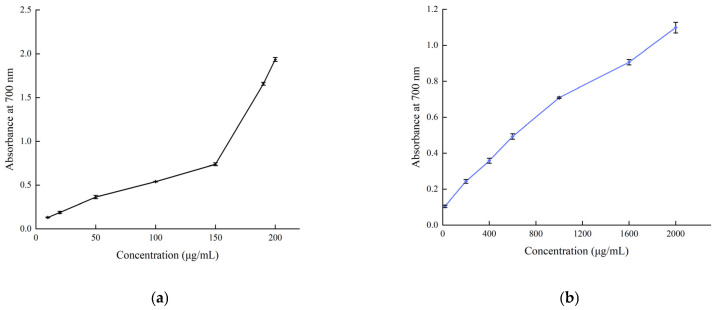
Total reducing power of (**a**) the ascorbic acid control and (**b**) *B. balsamifera* methanol extract.

The reducing power of *B. balsamifera* methanol extract and ascorbic acid control are shown in Figure 9. The reducing power of the methanol extract and ascorbic acid control showed an increasing trend, which was positively correlated with their mass concentrations. As shown in the figure, the absorbance value of ascorbic acid reached 1.935 ± 0.023 at a concentration of 200 μg/mL. The methanol extract reached its maximum value of 1.099 ± 0.03 at a concentration of 2000 μg/mL.

## 3. Discussion

This study analyzed the phytochemical composition and antioxidant potential of *B. balsamifera*, a plant with significant ethnobotanical relevance. An advanced UPLC–Q–Orbitrap HRMS technique was used to identify 31 compounds in the plant, including 18 compounds that have not been reported before. These compounds belong to various categories such as flavonoids, organic acids, quinones, and alkaloids. The identification of these compounds provides a chemical basis for understanding the medicinal value and mechanism of action of *B. balsamifera*. There are still many unknown peaks in the mass spectrum that have not been identified due to the lack of reference mass spectrometry data and substances for the corresponding compounds. Additional analysis is required to clarify these unknown compounds. Flavonoids formed the most abundant group among the identified compounds, with hemiphloin, taxifolin, rutin, and quercetin standing out in terms of their quantity. These flavonoids are well-documented for their potent antioxidant capabilities, which can be linked to the significant antioxidant activity observed in *B. balsamifera*. The detailed mass fragmentation patterns of these representative compounds could serve as a valuable reference for future phytochemical studies on this plant.

*B. balsamifera* has garnered significant interest in recent years in the quest for natural sources of antioxidants. Studies evaluating the antioxidant activity of this plant have primarily focused on its leaves. For instance, Ginting et al. investigated the antioxidant activity of n-hexane extract and its fractions from *B. balsamifera* leaves using a DPPH assay. However, the results showed that the antioxidant activity of the extracts was insignificant [55]. Jirakitticharoen et al. evaluated the antioxidant activity of *B. balsamifera* leaf extracts from different growth cycles using DPPH, ABTS, and FRAP assays. They found that the immature leaves of *B. balsamifera* were rich in quercetin and phenols and exhibited significant antioxidant activity [56]. Wang et al. compared and analyzed the antioxidant activity of essential oil components in the leaves of *B. balsamifera* picked at different times. They reported that the essential oils of *B. balsamifera* collected from October to December had higher antioxidant activity [57]. In this study, the antioxidant potential of methanol extract of *B. balsamifera* was assessed by evaluating its DPPH and ABTS free radical scavenging abilities, total antioxidative capacity, and reducing power. The results indicated that the methanol extract of the plant has considerable radical scavenging capabilities, thereby aligning with its traditional use as a natural antioxidant. There was a positive correlation observed between the antioxidant activities and the mass concentration of the methanol extract, as well as the polyphenol content, with a particular emphasis on flavonoids. Flavonoids are recognized for their strong antioxidant properties, thus contributing to the observed relationship. Although the potency was not as high as ascorbic acid, a benchmark antioxidant, it was noteworthy given the natural origin of the plant extract. This study contributes significantly to the existing body of knowledge about *B. balsamifera*, shedding light on its chemical diversity and antioxidant potential. These findings suggest that this plant could be a promising source of bioactive compounds for further pharmacological studies and possible drug development. Furthermore, the identification of flavonoids and other compounds could pave the way for the development of new antioxidant agents.

The study only focused on the chemical constituents and antioxidant activities of *B. balsamifera* methanol extract, which may not reflect the full spectrum of its bioactive components and pharmacological effects. However, the exploration of *B. balsamifera*’s therapeutic potential should not stop here. There are avenues for further investigations, such as in vivo antioxidant tests, and evaluations of other possible bioactivities. Additionally, the interactions between the identified compounds and their collective impact on the overall antioxidant activity of *B. balsamifera* could be an interesting focus for future research. This will not only provide a more holistic understanding of the plant’s therapeutic value but could also contribute to the optimization of extraction and formulation processes for therapeutic applications. Future research should also conduct phylogenetic and chemotaxonomic studies to clarify the relationship between *B. balsamifera* and other species in the genus *Blumea* or the family Asteraceae.

## 4. Materials and Methods

### 4.1. Plant Material and Extraction

*B. balsamifera* (L.) DC. was collected in July 2022 from the Yulin medicinal plant market in Guangxi, China. The plant material was authenticated by Associate Researcher Zheng Xilong from the School of Chinese Medicine Resources, Guangdong Pharmaceutical University. The dried aerial parts (moisture content not more than 10%) were ground using a DFY–300C swing pulverizer (Wenling LinDa Machinery Co., Ltd., Taizhou, China). The dry coarse powder of *B. balsamifera* was passed through a 50 mesh sieve (with a particle size ranging from 180 μm to 270 μm), and 10 g powder was accurately weighed and placed in a 50 mL flask. Methanol was added at a solid-to-liquid ratio of 1:50, and the mixture was extracted by ultrasound at a power of 300 W for 1 time, a temperature of 60 °C and time of 30 min, followed by centrifugation at 1500× *g* for 15 min. A volume of 200 μL of supernatant was taken and diluted to 1 mL with methanol. Then, it was filtered through a 0.22 μm membrane and stored at 4 °C before UPLC-Q-Orbitrap HRMS analysis. The remaining methanol extract was evaporated to obtain the crude methanol extract of *B. balsamifera* (BBME), which was stored at 4 °C for later use.

### 4.2. The Main Chemicals and Reagents

Analytical grade methanol (purchased from Da Mao Chemical Reagent Co., Ltd., Tianjin, China), chromatography-grade acetonitrile (obtained from Honeywell Trading (Shanghai) Co., Ltd., Shanghai, China), distilled water (from Watsons, Hong Kong, China), rutin, and quercetin (batch numbers: MUST–22022507 and MUST–22042012, respectively, were purchased from Chengdu Must Bio–Technology Co., Ltd., Chengdu, China), isoquercitrin, 1,3–dicaffeoylquinic acid, and chlorogenic acid (batch numbers: ZABH1013, AFBL1284, and AZ22011851, respectively, were obtained from Chengdu Alfa Biotechnology Co., Ltd., Chengdu, China). The purity of all reference compounds exceeded 98%, including ascorbic acid (Guangzhou Chemical Reagent Factory, Guangzhou, China) and 1,1–diphenyl–2–trinitrophenylhydrazine, 2,2–diazo–bis (3–ethylbenzothiazole–6–sulfonic acid) (Alsan Biotechnology Co., Guangzhou, China).

### 4.3. UPLC–Q–Orbitrap HRMS Analysis

#### 4.3.1. Instrumentation and Conditions

The chromatographic analysis was performed on a Vanquish Flex ultra-high-performance liquid chromatograph equipped with an Orbitrap Exploris 120 quadrupole-electrostatic orbitrap high-resolution mass spectrometer (Thermo Fisher Scientific, Waltham, MA, USA). Chromatographic separation was carried out on a Hypersil GOLD C_18_ column (100 mm × 2.1 mm, 1.9 μm) with a flow rate of 0.3 mL·min^–1^ and a column temperature of 35 °C. The injection volume was 2.00 μL. The mobile phase consisted of acetonitrile (A) and 0.1% formic acid aqueous solution (B), using a gradient elution program (0~5 min, 95%~75% B; 15~25 min, 75%~5% B; 25~27 min, 5% B; 27.001~30 min, 95% B). The mass spectrometer was operated in positive and negative ion-switching modes with the following conditions: heated electrospray ionization (HESI) source; spray voltage, 3.5 kV; capillary temperature, 325 °C; auxiliary gas temperature, 300 °C; mass scan range (*m*/*z*), 100~1500; sheath gas, auxiliary gas, and sweep gas were all nitrogen with flow rates of 50 L·min^–1^, 8 L·min^–1^, and 1 L·min^–1^, respectively, and; resolution, 60,000. The *B. balsamifera* sample was analyzed under the chromatographic and mass spectrometric conditions described above, and the total ion and MS^2^ chromatograms (Supplementary Figures S1–S33) in both positive and negative ion modes were obtained.

#### 4.3.2. Data Analysis and Compound Identification

The molecular formulas of the compounds corresponding to the chromatographic peaks were determined using the high-resolution exact mass values calculated by Xcalibur 4.1 software, the deviation between the measured and theoretical relative molecular masses (less than 5 × 10^–6^), and the isotopic abundance ratios. The possible chemical constituents were identified by comparing the *B. balsamifera* chemical composition database information (including English names, molecular formulas, and exact relative molecular masses), reference substances, database matching (mzVault and mzCloud databases, matching scores not less than 80), and fragmentation patterns, and confirmed through literature comparison.

### 4.4. Antioxidative Activity Evaluation

#### 4.4.1. DPPH Free Radical Scavenging Assay

The method is rapid and simple, and has been widely used to determine the antioxidant activity of biological samples, pure compounds and extracts in vitro [58,59]. The free-radical-scavenging ability against the 2,2–diphenyl–1–picryl–hydrazyl–hydrate (DPPH) was determined. The study determined the antioxidant activity of BBME utilizing the method developed by Li with slight modifications [60]. Different concentrations of BBME solutions, namely 3.33, 33.33, 66.67, 133.33, 200, 266.66, and 333 μg/mL were mixed with 2 mL of DPPH solution (2 mmol/L, dissolved in anhydrous ethanol). The resulting mixture was thoroughly blended and then reacted for 30 min at room temperature in the absence of light. The absorbance was measured at 517 nm with ascorbic acid as a positive control in the same 96-well plate. Each measurement was performed in triplicate. The percentage of DPPH-scavenging activity was calculated according to the equation:

$$\mathrm{DPPH}-\mathrm{scavenging}\;\mathrm{activity}\;(\%)=(\;1-\frac{A_{1}-A_{2}}{A_{0}}\;)\times 100\%$$

where A_1_ is the absorbance of the sample, A_2_ is the absorbance of the sample and ethanol without DPPH, and A_0_ is the absorbance of the control (the methanol in replace of the sample). The IC_50_ values of the samples were calculated by regressing the sample concentrations with the clearance rates.

#### 4.4.2. ABTS Free Radical Scavenging Assay

The 2,2′–azino–bis–3–ethylbenzthiazoline–6–sulphonic acid (ABTS) is a protonated radical. The method measures the antioxidant-scavenging ability of the ABTS radical generated by potassium persulfate in the aqueous phase. It has a characteristic maximum absorbance value at 734 nm and decreases with the scavenging of protonated radicals [61]. The antioxidant activity of the BBME was evaluated using the ABTS free radical scavenging assay, with slight modifications from a previously reported method [62]. Briefly, ABTS (7 mM) was reacted with potassium persulfate (2.45 mM) and allowed to stand at room temperature for 12 h in the dark. The ABTS radical cation (ABTS^•+^) was diluted with ethanol to give an absorbance of 0.70 ± 0.02 at 734 nm. An amount of 2 mL of the BBME solutions of different concentrations (3.33, 16.67, 33.33, 50, 100, 200 and 300 μg/mL) was mixed with 2 mL of ABTS^•+^ solution and the mixture was left to stand in darkness for 30 min. Absorbance was measured at 734 nm. Ascorbic acid was used as a positive control. The percentage inhibition of ABTS^•+^ radical was calculated using the following equation:

$$\mathrm{ABTS}-\mathrm{scavenging}\;\mathrm{activity}\;(\%)=(\;1-\frac{A_{1}-A_{2}}{A_{0}}\;)\times 100\%$$

where A_1_ represents the absorbance of the sample, A_2_ represents the absorbance of the sample and ethanol without ABTS, and A_0_ represents the absorbance of the control (the methanol in replace of the sample). The IC_50_ values of the samples were calculated by regressing the sample concentrations with the clearance rates.

#### 4.4.3. Total Antioxidant Capacity Assay

The total antioxidant capacity of the BBME was measured using the phosphomolybdenum method [63]. The method is based on the reduction in Mo(VI)–Mo(V) by the sample tested and the subsequent formation of green phosphate–Mo(V) complex with a maximum absorption at 695 nm. In brief, 1 mL of the BBME solutions of different concentrations (20, 50, 100, 200, 300, and 400 μg/mL) (with methanol as the blank) were mixed with 1 mL of the reagent solution (0.6 M sulfuric acid, 28 mM sodium phospha–te, and 4 mM ammonium molybdate). The tubes were capped and incubated in a water bath at 90 °C for 90 min. After cooling, the absorbance of each solution was measured at 695 nm against a blank. The antioxidant capacity was expressed as the number of equivalents of ascorbic acid.

#### 4.4.4. Reducing Power Assay

The reducing power of an active substance can be an important indicator of its potential antioxidant activity. The absorbance measured at 700 nm was used to demonstrate the reducing power. An increase in the absorbance of the measured sample indicates an increase in the reducing power of the sample tested [64]. The total reducing power of the BBME was evaluated by the method of Oyaizu [65]. In brief, 2 mL of the BBME solutions of different concentrations (20, 200, 400, 600, 1000, 1600, and 2000 μg/mL, with methanol as the blank) was mixed with 2 mL of phosphate buffer (0.2 M, pH 6.6) and 2 mL of 1% potassium ferricyanide. The mixture was then incubated at 50 °C for 20 min. After the mixture was cooled, 2 mL of 10% trichloroacetic acid was added, and the mixture was centrifuged at 3000 rpm for 10 min. The upper layer of the solution (2 mL) was mixed with 2 mL of distilled water and 0.5 mL of 0.1% ferric chloride. The absorbance was measured at 700 nm against a blank solution. Ascorbic acid was used as the standard.

### 4.5. Statistical Analysis

All tests were conducted in triplicate. Data were presented as mean ± SD. Statistical analysis was performed using the SPSS software (Version: 20.0, Armonk, NY, USA). The data were subjected to one-way analysis of variance (ANOVA), and the significant differences were evaluated using Duncan’s test (*p* < 0.05).

## Figures and Tables

**Table 1:** Identification of chemical constituents from *B. balsamifera*.

NO.	RT/min	Compound	Molecular Formula	Ion Mode	*m*/*z*	Error/ppm	Compound Types	References
1	0.85	*α*,*α*–trehalose	C_12_H_22_O_11_	[M−H]^−^	341.1085	−0.25	saccharide	[19]
2	0.86	*D*–(−)–fructose ^∆^	C_6_H_12_O_6_	[M−H]^−^	179.0559	−1.42	saccharide	[20]
3	2.12	gentisic acid 5–O–β–glucoside	C_13_H_16_O_9_	[M−H]^−^	315.0719	−0.91	Phenolic glycosides	[21]
4	5.89	caffeic acid ^∆^	C_9_H_8_O_4_	[M−H]^−^	179.0348	−1.07	organic acids	[22]
5	7.27	tuberonic acid glucoside ^∆^	C_18_H_28_O_9_	[M−H]^−^	387.1658	−0.73	organic acids	[23]
6	10.15	hemiphloin ^∆^	C_21_H_22_O_10_	[M−H]^−^	433.1154	−0.64	flavonoids	[24]
7	10.33	taxifolin	C_15_H_12_O_7_	[M−H]^−^	303.0509	−0.49	flavonoids	[17]
8	10.37	rutin *	C_27_H_30_O_16_	[M−H]^−^	609.1456	−0.73	flavonoids	[25]
9	11.08	quercetin–3β–D–glucoside	C_21_H_20_O_12_	[M−H]^−^	463.0876	−0.97	flavonoids	[26]
10	11.08	quercetin *	C_15_H_10_O_7_	[M+H]^+^	303.0500	0.3	flavonoids	[25]
11	12.02	quercetin–3–arabinoside ^∆^	C_20_H_18_O_11_	[M−H]^−^	433.0772	−0.67	flavonoids	[27]
12	12.05	cynaroside ^∆^	C_21_H_20_O_11_	[M−H]^−^	447.0928	−0.8	flavonoids	[28]
13	12.66	1,3–dicaffeoylquinic acid *	C_25_H_24_O_12_	[M−H]^−^	515.1190	−0.89	organic acids	[29]
14	12.66	chlorogenic acid *	C_16_H_18_O_9_	[M−H]^−^	353.0878	−0.36	organic acids	[30]
15	13.02	isorhamnetin–3–O–glucoside ^∆^	C_22_H_22_O_12_	[M−H]^−^	477.1034	−0.82	flavonoids	[31]
16	13.42	4–oxo–5–phenylpentanoic acid ^∆^	C_11_H_12_O_3_	[M+H]^+^	193.0859	0.06	organic acids	[32]
17	16.238	eriodictyol	C_15_H_12_O_6_	[M−H]^−^	287.0559	−0.66	flavonoids	[33]
18	17.87	padmatin	C_16_H_14_O_7_	[M−H]^−^	317.0666	−0.22	flavonoids	[34]
19	17.97	isorhamnetin * ^∆^	C_16_H_12_O_7_	[M−H]^−^	315.0509	−0.17	flavonoids	[35]
20	18.43	naringenin ^∆^	C_15_H_12_O_5_	[M−H]^−^	271.0611	−0.26	flavonoids	[36]
21	19.13	7–O–methylaromadendrin ^∆^	C_16_H_14_O_6_	[M+H]^+^	303.0865	0.47	flavonoids	[37]
22	19.36	aurantio–obtusin ^∆^	C_17_H_14_O_7_	[M+H]^+^	331.0815	0.16	quinones	[38]
23	19.36	fraxetin	C_10_H_8_O_5_	[M+H]^+^	209.0445	0.14	coumarins	[39]
24	19.76	hematoxylin	C_16_H_14_O_6_	[M−H]^−^	301.0716	−0.54	phenols	[40]
25	20.67	sakuranetin ^∆^	C_16_H_14_O_5_	[M+H]^+^	287.0915	0.16	flavonoids	[41]
26	21.52	aurantiamide acetate ^∆^	C_27_H_28_N_2_O_4_	[M+H]^+^	445.2121	−0.05	alkaloids	[42]
27	23.03	cryptotanshinone ^∆^	C_19_H_20_O_3_	[M+H]^+^	297.1485	−0.03	quinones	[43]
28	23.17	nootkatone ^∆^	C_15_H_22_O	[M+H]^+^	219.1743	−0.12	sesquiterpenes	[44]
29	23.29	N–phenyl–1–naphthylamine ^∆^	C_16_H_13_N	[M+H]^+^	220.1121	0.07	alkaloids	[45]
30	24.06	tanshinone IIA	C_19_H_18_O_3_	[M+H]^+^	295.2270	−0.12	quinones	[46]
31	24.70	citroflex A–4 ^∆^	C_20_H_34_O_8_	[M+Na]^+^	425.2145	−0.13	lipid	[47]

* Indicates that these compounds were identified by reference substance comparison. ^∆^ Indicates the first identification of these compounds from *B. balsamifera*.

**Table 2:** Antioxidant activities of *B. balsamifera* methanol extract expressed as IC_50_ values (μg/mL) for DPPH, ABTS radical scavenging activity.

Samples	DPPH IC_50_ (μg/mL)	ABTS IC_50_ (μg/mL)
*B. balsamifera*	105.1 ± 0.503	12.49 ± 0.341
Ascorbic acid	0.98 ± 0.073	1.774 ± 0.036

## Data Availability

Not applicable.

## Supplementary Materials

The following supporting information can be downloaded at: https://www.mdpi.com/article/10.3390/molecules28114504/s1, Figures S1–S31: MS^2^ fragmentation spectra (CID) of compounds; Figures S32 and S33: TICs of *B. balsamifera* in negative and positive ion mode.

## References

[1] Diniz Vilela D., Gomes Peixoto L., Teixeira R.R., Belele Baptista N., Carvalho Caixeta D., De Souza A.V., Machado H.L., Pereira M.N., Sabino–Silva R., Espindola F.S. (2016). The Role of Metformin in Controlling Oxidative Stress in Muscle of Diabetic Rats. Oxid. Med. Cell Longev..

[2] Kasote D.M., Katyare S.S., Hegde M.V., Bae H. (2015). Significance of antioxidant potential of plants and its relevance to therapeutic applications. Int. J. Biol. Sci..

[3] Embuscado M.E. (2015). Spices and herbs: Natural sources of antioxidants–a mini review. J. Funct. Foods.

[4] Xu D.P., Li Y., Meng X., Zhou T., Zhou Y., Zheng J., Zhang J.J., Li H.B. (2017). Natural antioxidants in foods and medicinal plants: Extraction, assessment and resources. Int. J. Mol. Sci..

[5] Editorial Committee of Flora of China, Chinese Academy of Sciences (1979). Flora of China.

[6] Pang Y., Wang D., Fan Z., Chen X., Yu F., Hu X., Wang K., Yuan L. (2014). *Blumea balsamifera*–a phytochemical and pharmacological review. Molecules.

[7] Widhiantara I.G., Jawi I.M. (2021). Phytochemical composition and health properties of Sembung plant (*Blumea balsamifera*): A review. Vet. World.

[8] Madjos G.G., Ramos K.P. (2021). Ethnobotany, systematic review and field mapping on folkloric medicinal plants in the Zamboanga Peninsula, Mindanao, Philippines. J. Compl. Med. Res..

[9] Jiang Z.L., Zhou Y., Ge W.C., Yuan K. (2014). Phytochemical compositions of volatile oil from *Blumea balsamifera* and their biological activities. Pharmacogn. Mag..

[10] Xiong Y., Yi P., Li Y., Gao R., Chen J., Hu Z., Lou H., Du C., Zhang J., Zhang Y. (2022). New sesquiterpeniod esters form *Blumea balsamifera* (L.) DC. and their anti–influenza virus activity. Nat. Prod. Res..

[11] Huang X.L., Wang D.W., Liu Y.Q., Cheng Y.X. (2022). Diterpenoids from *Blumea balsamifera* and Their Anti–Inflammatory Activities. Molecules.

[12] Osaki N., Koyano T., Kowithayakorn T., Hayashi M., Komiyama K., Ishibashi M. (2005). Sesquiterpenoids and Plasmin–Inhibitory Flavonoids from *Blumea balsamifera*. J. Nat. Prod..

[13] Chen M., Qin J.J., Fu J.J., Hu X.J., Liu X.H., Zhang W.D., Jin H.Z. (2010). Blumeaenes A–J, sesquiterpenoid esters from *Blumea balsamifera* with NO inhibitory activity. Planta Med..

[14] Xu J., Jin D.Q., Liu C., Xie C., Guo Y., Fang L. (2012). Isolation, Characterization, and NO Inhibitory Activities of Sesquiterpenes from *Blumea balsamifera*. J. Agric. Food Chem..

[15] Li T., Zeng H., Zeng Y., Zhang X., Ren Y., Gao Y., Huang Q., Tan J. (2021). Characterization of the bioactive compounds with efficacy against gout in Guizhi Shaoyao Zhimu Decoction by UHPLC–Q–Orbitrap HRMS combined with network pharmacological analysis. Arab. J. Chem..

[16] Liu R., Su C., Xu Y., Shang K., Sun K., Li C., Lu J. (2020). Identifying potential active components of walnut leaf that action diabetes mellitus through integration of UHPLC–Q–Orbitrap HRMS and network pharmacology analysis. J. Ethnopharmacol..

[17] Liu R., Zhao Z., Dai S., Che X., Liu W. (2019). Identification and Quantification of Bioactive Compounds in *Diaphragma juglandis* Fructus by UHPLC–Q–Orbitrap HRMS and UHPLC–MS/MS. J. Agric. Food Chem..

[18] Yang L., Gao S., Su Z., Qin X., Li Z. (2021). Identification of the constituents and the cancer–related targets of the fruit of *Solanum nigrum* based on molecular docking and network pharmacology. J. Pharm. Biomed. Anal..

[19] Tao Y., Rossez Y., Bortolus C., Duma L., Dubar F., Merlier F. (2023). Simultaneous Quantification of Trehalose and Trehalose 6–Phosphate by Hydrophilic Interaction Chromatography/Electrospray Accurate Mass Spectrometry with Application in Non–Targeted Metabolomics. Molecules.

[20] Li N., Wang J., Wang B., Huang S., Hu J., Yang T., Asmutola P., Lan H.Y., Yu Q.H. (2021). Identification of the carbohydrate and organic acid metabolism genes responsible for Brix in tomato fruit by transcriptome and metabolome analysis. Front. Genet..

[21] Kacem N., Hay A.E., Marston A., Zellagui A., Rhouati S., Hostettmann K. (2012). Antioxidant compounds from *Algerian Convolvulus* tricolor (Convolvulaceae) seed husks. Nat. Prod. Commun..

[22] Keckes S., Gasic U., Velickovic T.C., Milojkovic–Opsenica D., Natic M., Tesic Z. (2013). The determination of phenolic profiles of *Serbian unifloral* honeys using ultra–high–performance liquid chromatography/high resolution accurate mass spectrometry. Food Chem..

[23] Silva M.F.S., Silva L.M.A., Quintela A.L., Dos Santos A.G., Silva F.A.N., de Oliveira F.D.C.E., Alves Filho E.G., De Brito E.S., Canuto K.M., Pessoa C. (2019). UPLC–HRMS and NMR applied in the evaluation of solid–phase extraction methods as a rational strategy of dereplication of *Phyllanthus* spp. aiming at the discovery of cytotoxic metabolites. J. Chromatogr. B.

[24] Nunes Alves Paim L.F., dos Santos P.R., Patrocinio Toledo C.A., Minello L., Lima da Paz J.R., Castro Souza V., Salvador M., Moura S. (2022). Four almost unexplored species of *Brazilian Connarus* (Connaraceae): Chemical composition by ESI–QTof–MS/MS–GNPS and a pharmacologic potential. Phytochem. Anal..

[25] Chen Y.Q., Yu H.L., Wu H., Pan Y.Z., Wang K.L., Jin Y.P., Zhang C.C. (2015). Characterization and quantification by LC–MS/MS of the chemical components of the heating products of the flavonoids extract in *Pollen Typhae* for transformation rule exploration. Molecules.

[26] He X.J., Liu R.H. (2008). Phytochemicals of apple peels: Isolation, structure elucidation, and their antiproliferative and antioxidant activities. J. Agric. Food Chem..

[27] Zhang C.T., Ma Y.L., Zhao Y.X., Hong Y.Q., Cai S.B., Pang M.J. (2018). Phenolic composition, antioxidant and pancreatic lipase inhibitory activities of Chinese sumac (*Rhus chinensis* Mill.) fruits extracted by different solvents and interaction between myricetin–3–O–rhamnoside and quercetin–3–O–rhamnoside. Int. J. Food Sci. Tech..

[28] Jerman Klen T., Golc Wondra A., Vrhovsek U., Mozetic Vodopivec B. (2015). Phenolic Profiling of Olives and Olive Oil Process–Derived Matrices Using UPLC–DAD–ESI–QTOF–HRMS Analysis. J. Agric. Food Chem..

[29] Duthen S., Gadea A., Trempat P., Boujedaini N., Fabre N. (2022). Comparison of the Phytochemical Variation of Non–Volatile Metabolites within Mother Tinctures of Arnica montana Prepared from Fresh and Dried Whole Plant Using UHPLC–HRMS Fingerprinting and Chemometric Analysis. Molecules.

[30] Vereshchagina Y.V., Bulgakov V.P., Grigorchuk V.P., Rybin V.G., Veremeichik G.N., Tchernoded G.K., Gorpenchenko T.Y., Koren O.G., Phan N.H.T., Minh N.T. (2014). The rolC gene increases caffeoylquinic acid production in transformed artichoke cells. Appl. Microbiol. Biot..

[31] Lin L.Z., Sun J.H., Chen P., Harnly J. (2011). UHPLC–PDA–ESI/HRMS/MSn analysis of anthocyanins, flavonol glycosides, and hydroxycinnamic acid derivatives in red mustard greens (*Brassica juncea* Coss Variety). J. Agric. Food Chem..

[32] Sumarah M.W., Puniani E., Sorensen D., Blackwell B.A., Miller J.D. (2010). Secondary metabolites from anti–insect extracts of endophytic fungi isolated from *Picea rubens*. Phytochemistry.

[33] Shu Y.S., Liu Z.L., Zhao S.Y., Song Z.Q., He D., Wang M.L., Zeng H.L., Lu C., Lu A.P., Liu Y.Y. (2017). Integrated and global pseudotargeted metabolomics strategy applied to screening for quality control markers of Citrus TCMs. Anal. Bioanal. Chem..

[34] Mohammed H.A., Almahmoud S.A., El–Ghaly E.M., Khan F.A., Emwas A., Jaremko M., Almulhim F., Khan R.A., Ragab E.A. (2022). Comparative Anticancer Potentials of Taxifolin and Quercetin Methylated Derivatives against HCT–116 Cell Lines: Effects of O–Methylation on Taxifolin and Quercetin as Preliminary Natural Leads. ACS Omega.

[35] Campone L., Celano R., Lisa Piccinelli A., Pagano I., Carabetta S., Sanzo R.D., Russo M., Ibanez E., Cifuentes A., Rastrelli L. (2018). Response surface methodology to optimize supercritical carbon dioxide/co–solvent extraction of brown onion skin by–product as source of nutraceutical compounds. Food Chem..

[36] Funari C.S., Passalacqua T.G., Rinaldo D., Napolitano A., Festa M., Capasso A., Piacente S., Pizza C., Young M.C.M., Durigan G. (2011). Interconverting flavanone glucosides and other phenolic compounds in *Lippia salviaefolia* Cham. ethanol extracts. Phytochemistry.

[37] Trimech I., Weiss E.K., Chedea V.S., Marin D., Detsi A., Ioannou E., Roussis V., Kefalas P. (2014). Evaluation of Anti–oxidant and Acetylcholinesterase Activity and Identification of Polyphenolics of the Invasive Weed *Dittrichia viscosa*. Phytochem. Anal..

[38] Zhao Y.Y., Cao J.L., Zhao J.J., Wei P.H., Wu R., Zhang J.X., Wan L. (2022). Chemical analysis of Chrysosplenium from different species by UPLC–Q exactive orbitrap HRMS and HPLC–DAD. J. Pharmaceut. Biomed..

[39] Zhou L., Kang J., Fan L., Ma X.C., Zhao H.Y., Han J., Wang B.R., Guo D.A. (2008). Simultaneous analysis of coumarins and secoiridoids in Cortex Fraxini by high–performance liquid chromatography–diode array detection–electrospray ionization tandem mass spectrometry. J. Pharmaceut. Biomed..

[40] Ahram M., Flaig M.J., Gillespie J.W., Duray P.H., Linehan W.M., Ornstein D.K., Niu S., Zhao Y.M., Petricoin E.F., Emmert-Buck M.R. (2003). Evaluation of ethanol–fixed, paraffin–embedded tissues for proteomic applications. Proteomics.

[41] Lopez-Gutierrez N., Romero-Gonzalez R., Garrido Frenich A., Martinez Vidal J.L. (2014). Identification and quantification of the main isoflavones and other phytochemicals in soy based nutraceutical products by liquid chromatography–orbitrap high resolution mass spectrometry. J. Chromatogr. A.

[42] Kongstad K.T., Wubshet S.G., Kjellerup L., Winther A.L., Staerk D. (2015). Fungal plasma membrane H+–ATPase inhibitory activity of o–hydroxybenzylated flavanones and chalcones from *Uvaria chamae* P. Beauv. Fitoterapia.

[43] Sairafianpour M., Christensen J., Strk D., Budnik B.A., Kharazmi A., Bagherzadeh K., Jaroszewski J.W. (2001). Leishmanicidal, antiplasmodial, and cytotoxic activity of novel diterpenoid 1,2–quinones from *Perovskia abrotanoides*: New source of tanshinones. J. Nat. Prod..

[44] Xu H.B., Ma Y.B., Huang X.Y., Geng C.A., Wang H., Zhao Y., Yang T.H., Chen X.L., Yang C.Y., Zhang X.M. (2015). Bioactivity–guided isolation of anti–hepatitis B virus active sesquiterpenoids from the traditional Chinese medicine: Rhizomes of *Cyperus rotundus*. J. Ethnopharmacol..

[45] Ungeheuer F., van Pinxteren D., Vogel A.L. (2021). Identification and source attribution of organic compounds in ultrafine particles near Frankfurt International Airport. Atmos. Chem. Phys..

[46] Ming Q.L., Han T., Li W.C., Zhang Q.Y., Zhang H., Zheng C.J., Huang F., Rahman K., Qin L.P. (2012). Tanshinone IIA and tanshinone I production by Trichoderma atroviride D16, an endophytic fungus in *Salvia miltiorrhiza*. Phytomedicine.

[47] Tehrani M.W., Newmeyer M.N., Rule A.M., Prasse C. (2021). Characterizing the Chemical Landscape in Commercial E–Cigarette Liquids and Aerosols by Liquid Chromatography–High–Resolution Mass Spectrometry. Chem. Res. Toxicol..

[48] Cuyckens F., Claeys M. (2004). Mass spectrometry in the structural analysis of flavonoids. J. Mass Spectrom..

[49] Bai Y., Song F., Chen M., Xing J.P., Liu Z.Q. (2004). Characterization of the rutin–metal complex by electrospray ionization tandem mass spectrometry. Anal. Sci..

[50] Guo X.F., Yue Y.D., Tang F., Wang J., Yao X., Sun J. (2013). A comparison of C–glycosidic flavonoid isomers by electrospray ionization quadrupole time–of–flight tandem mass spectrometry in negative and positive ion mode. Int. J. Mass Spectrom.

[51] Yuan T., Guo X.F., Shao S.Y., An R.M., Wang J., Sun J. (2021). Characterization and identification of flavonoids from *Bambusa chungii* leaves extract by UPLC–ESI–Q–TOF–MS/MS. Acta. Chromatogr..

[52] Jaiswal R., Müller H., Müller A., Müller A., Karar M.G.E., Kuhnert N. (2014). Identification and characterization of chlorogenic acids, chlorogenic acid glycosides and flavonoids from *Lonicera henryi* L.(Caprifoliaceae) leaves by LC–MSn. Phytochemistry.

[53] Li J.R., Li D.X., Li L., Deng W.L., Ding L.S., Xu H.X., Zhou Y. (2014). Simultaneous quantification of salvianolic acid B and tanshinone IIA of salvia tropolone tablets by UPLC–MRM–MS/MS for pharmacokinetic studies. Acta. Chromatogr..

[54] Boonen J., Sharma V., Dixit V.K., Burvenich C., Spiegeleer B.D. (2012). LC–MS N–alkylamid profiling of an ethanolic *Anacyclus pyrethrum* root extract. Planta. Med..

[55] Ginting B., Maulana I., Yahya M., Saidi N., Murniana M., Hasballah K., Maulidna M., Rawati S. (2022). Antioxidant and antiproliferative activities of n-hexane extract and its fractions from *Blumea balsamifera* L. leaves. J. Adv. Pharm. Technol. Res..

[56] Jirakitticharoen S., Wisuitiprot W., Jitareerat P., Wongs-Aree C. (2022). Phenolics, antioxidant and antibacterial activities of immature and mature *Blumea balsamifera* leaf extracts eluted with different solvents. J. Trop. Med..

[57] Wang Y.H., Zhang Y.R. (2020). Variations in compositions and antioxidant activities of essential oils from leaves of Luodian *Blumea balsamifera* from different harvest times in China. PLoS ONE.

[58] Xu Z., Cai Y., Ma Q., Zhao Z., Yang D., Xu X. (2021). Optimization of extraction of bioactive compounds from *Baphicacanthus cusia* leaves by hydrophobic deep eutectic solvents. Molecules.

[59] Ozturk B., Parkinson C., Gonzalez–Miquel M. (2018). Extraction of polyphenolic antioxidants from orange peel waste using deep eutectic solvents. Sep. Purif. Technol..

[60] Li Z., Li Q. (2022). Ultrasonic–Assisted Efficient Extraction of Coumarins from *Peucedanum decursivum* (Miq.) Maxim Using Deep Eutectic Solvents Combined with an Enzyme Pretreatment. Molecules.

[61] Mathew S., Abraham T.E. (2006). In vitro antioxidant activity and scavenging effects of *Cinnamomum verum* leaf extract assayed by different methodologies. Food Chem. Toxicol..

[62] Li X.C., Lin J., Gao Y.X., Han W.J., Chen D.F. (2012). Antioxidant activity and mechanism of Rhizoma *Cimicifugae*. Chem. Cent. J..

[63] Prieto P., Pineda M., Aguilar M. (1999). Spectrophotometric quantitation of antioxidant capacity through the formation of phosphomolybdenum complex: Specific application to determination of vitamin E. Anal. Biochem..

[64] Tsai S.Y., Huang S.J., Mau J.L. (2006). Antioxidant properties of hot water extracts from *Agrocybe cylindracea*. Food Chem..

[65] Oyaizu M. (1986). Studies on product of browning reaction prepared from glucose amine. Jpn. J. Nutr..

