# Supplementary material for: Identification of Chemical Constituents in Blumea balsamifera Using UPLC–Q–Orbitrap HRMS and Evaluation of Their Antioxidant Activities
Source: Molecules. 2023 Jun 1;28(11):4504. doi: 10.3390/molecules28114504 (PMC10254267; doi:10.3390/molecules28114504)
Supplement: Supplementary file 1 [file molecules-28-04504-s001.zip › molecules-2435701-supplementary.pdf]

Identification of Chemical Constituents in *Blumea balsamifera* Using UPLC–Q–Orbitrap HRMS and Evaluation of Their Antioxidant Activities

Liping Dai [1,2], Shengnan Cai [1,2], Dake Chu [2], Rui Pang [2], Jianhao Deng [2], Xilong Zheng [1,*]

and Wei Dai [2,*]

Figure S1. MS^2 fragmentation spectra (CID) of *α*,*α*–trehalose
Figure S2. MS^2 fragmentation spectra (CID) of D–(–)–fructose
Figure S3. MS^2 fragmentation spectra (CID) of gentisic acid 5–O–*β*–glucoside
Figure S4. MS^2 fragmentation spectra (CID) of caffeic acid
Figure S5. MS^2 fragmentation spectra (CID) of tuberonic acid glucoside
Figure S6. MS^2 fragmentation spectra (CID) of hemiphloin
Figure S7. MS^2 fragmentation spectra (CID) of taxifolin
Figure S8. MS^2 fragmentation spectra (CID) of rutin
Figure S9. MS^2 fragmentation spectra (CID) of quercetin–3*β*–D–glucoside
Figure S10. MS^2 fragmentation spectra (CID) of quercetin
Figure S11. MS^2 fragmentation spectra (CID) of quercetin–3–Arabinoside
Figure S12. MS^2 fragmentation spectra (CID) of cynaroside
Figure S13. MS^2 fragmentation spectra (CID) of 1,3–Dicaffeoylquinic acid
Figure S14. MS^2 fragmentation spectra (CID) of chlorogenic acid
Figure S15. MS^2 fragmentation spectra (CID) of isorhamnetin–3–O–glucoside
Figure S16. MS^2 fragmentation spectra (CID) of 4–oxo–5–phenylpentanoic acid
Figure S17. MS^2 fragmentation spectra (CID) of eriodictyol
Figure S18. MS^2 fragmentation spectra (CID) of padmatin
Figure S19. MS^2 fragmentation spectra (CID) of isorhamnetin
Figure S20. MS^2 fragmentation spectra (CID) of naringenin
Figure S21. MS^2 fragmentation spectra (CID) of 7–O–Methylaromadendrin
Figure S22. MS^2 fragmentation spectra (CID) of aurantio–obtusin
Figure S23. MS^2 fragmentation spectra (CID) of fraxetin
Figure S24. MS^2 fragmentation spectra (CID) of hematoxylin
Figure S25. MS^2 fragmentation spectra (CID) of sakuranetin
Figure S26. MS^2 fragmentation spectra (CID) of aurantiamide acetate
Figure S27. MS^2 fragmentation spectra (CID) of cryptotanshinone
Figure S28. MS^2 fragmentation spectra (CID) of nootkatone
Figure S29. MS^2 fragmentation spectra (CID) of N–Phenyl–1–naphthylamine
Figure S30. MS^2 fragmentation spectra (CID) of tanshinone IIA
Figure S31. MS^2 fragmentation spectra (CID) of citroflex A–4
Figure S32. TICs of *Blumea balsamifera* (L.) DC. in negative ion mode
Figure S33. TICs of *Blumea balsamifera* (L.) DC. in positive ion mode

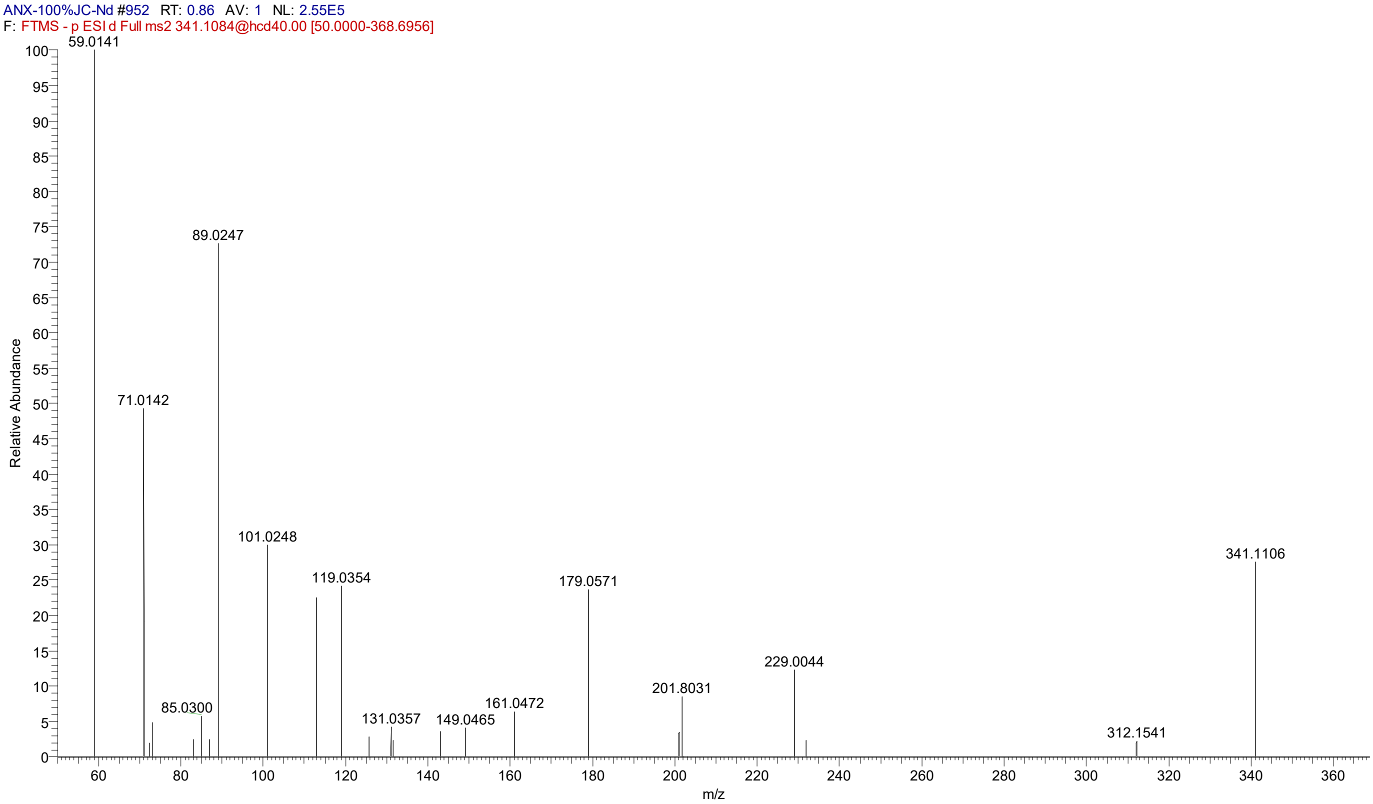

Figure S1. MS² fragmentation spectra (CID) of *α,α*–trehalose

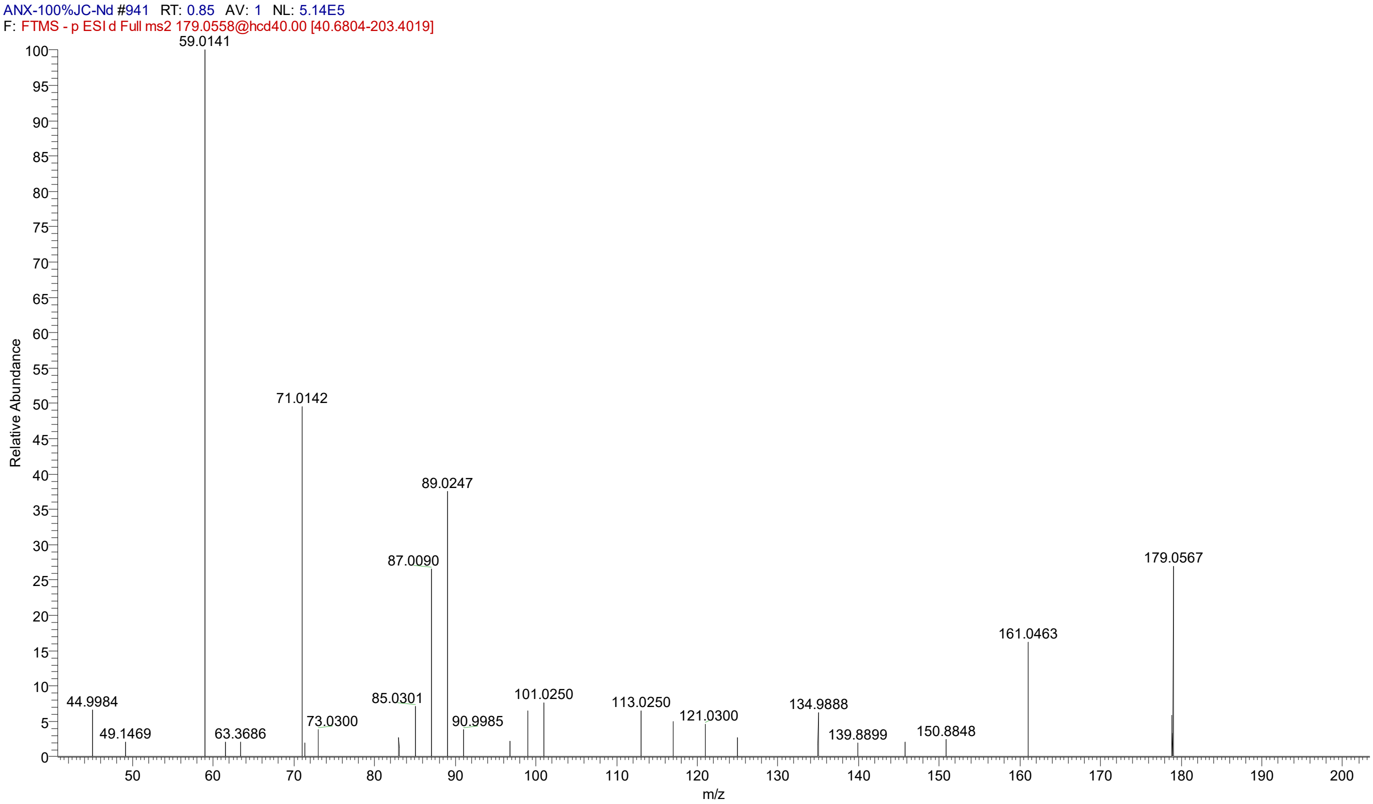

Figure S2. MS² fragmentation spectra (CID) of D–(–)–fructose

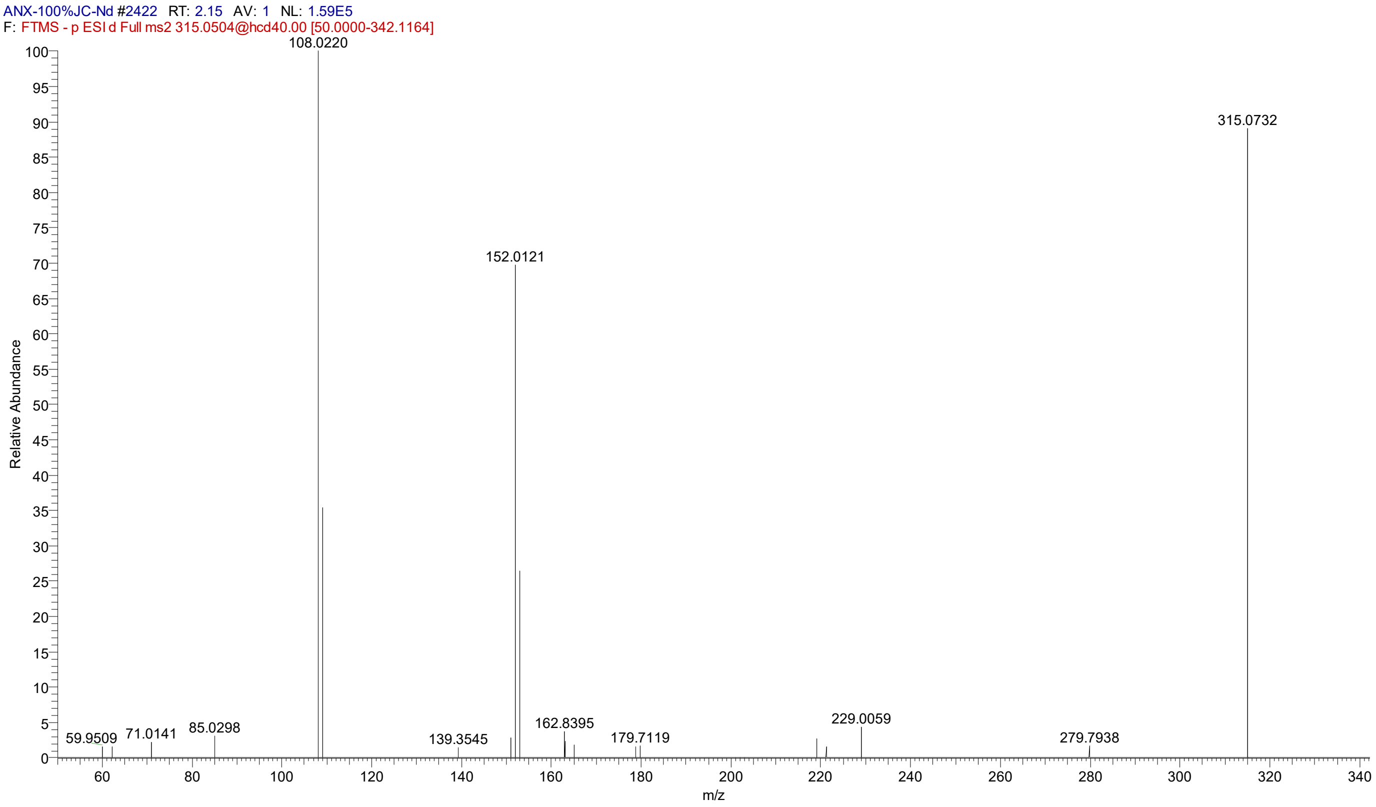

Figure S3. MS^2 fragmentation spectra (CID) of gentisic acid 5–O–*β*–glucoside

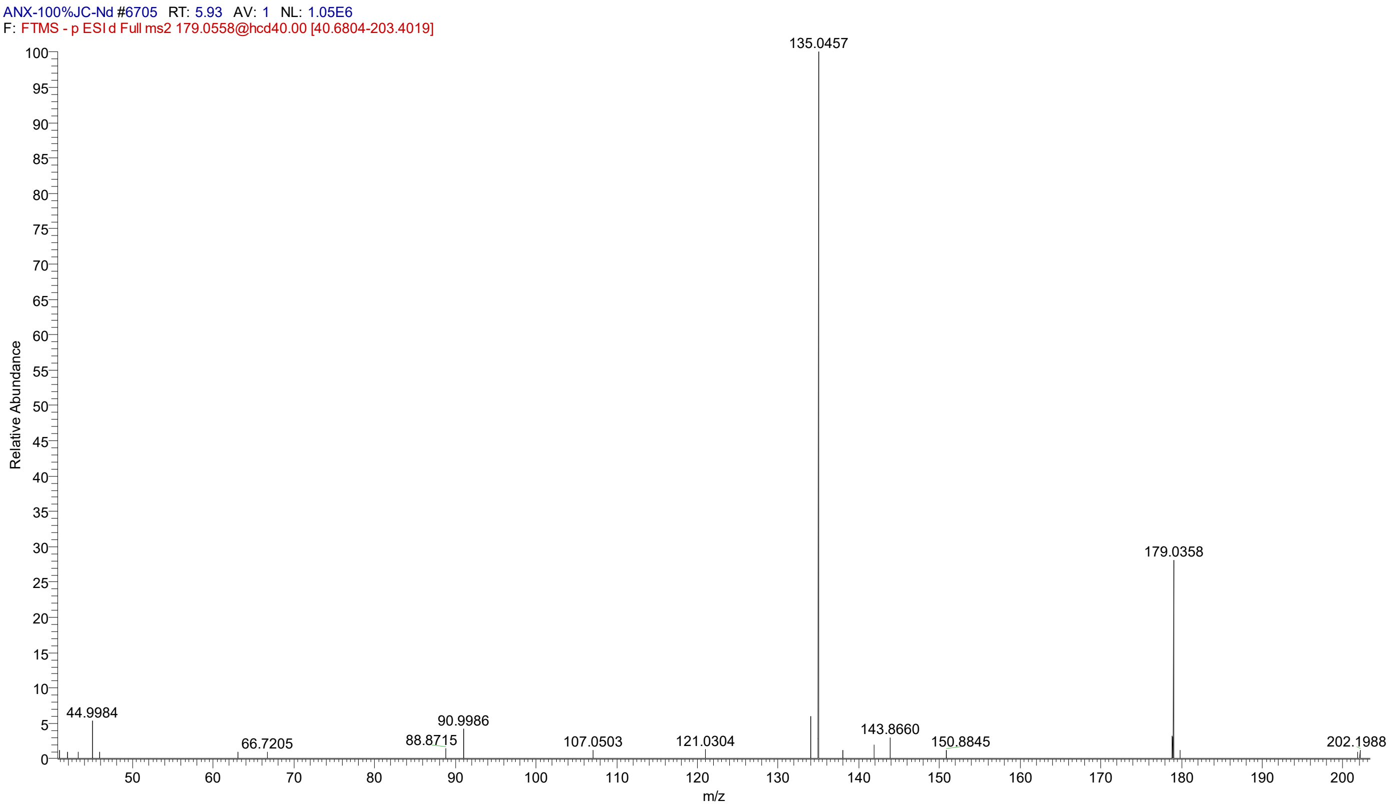

Figure S4. MS^2 fragmentation spectra (CID) of caffeic acid

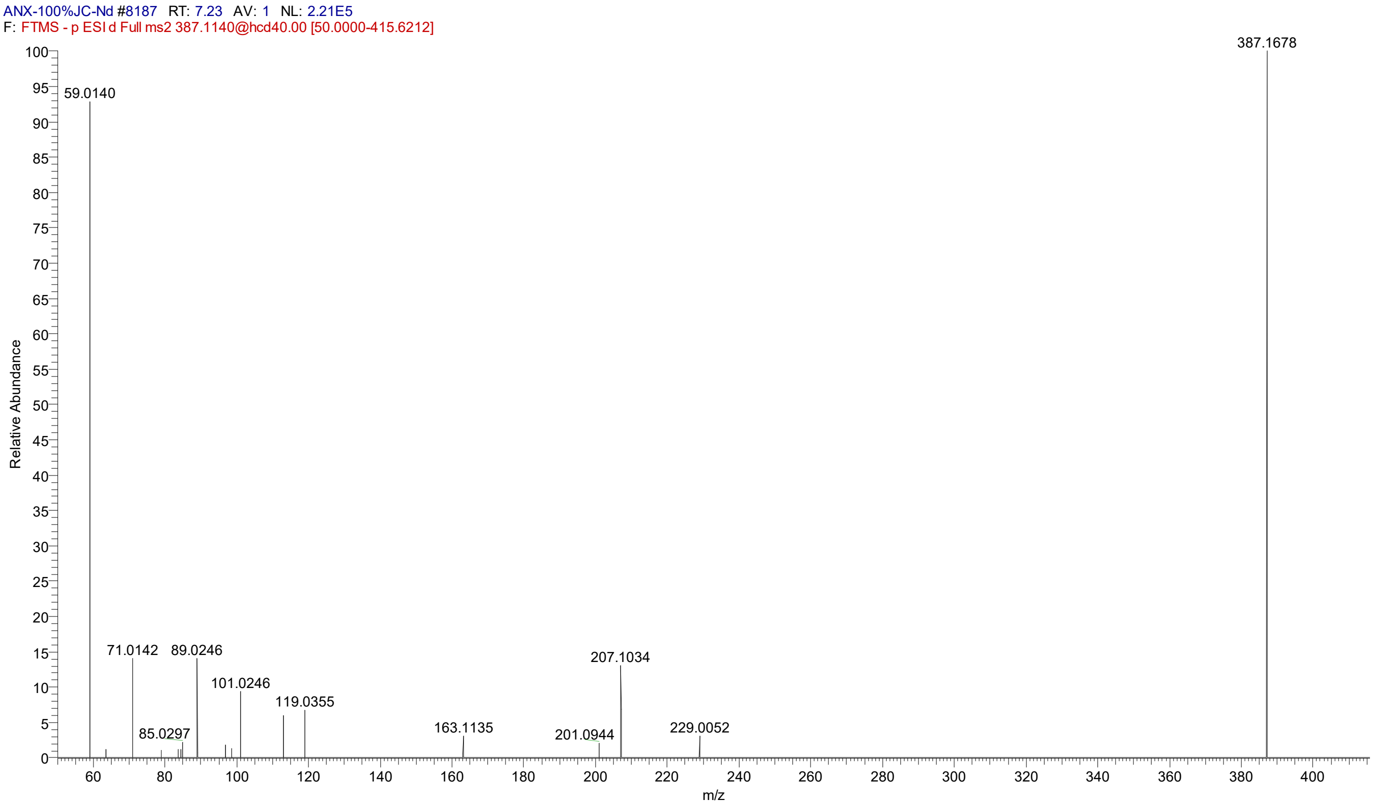

Figure S5. MS² fragmentation spectra (CID) of tuberonic acid glucoside

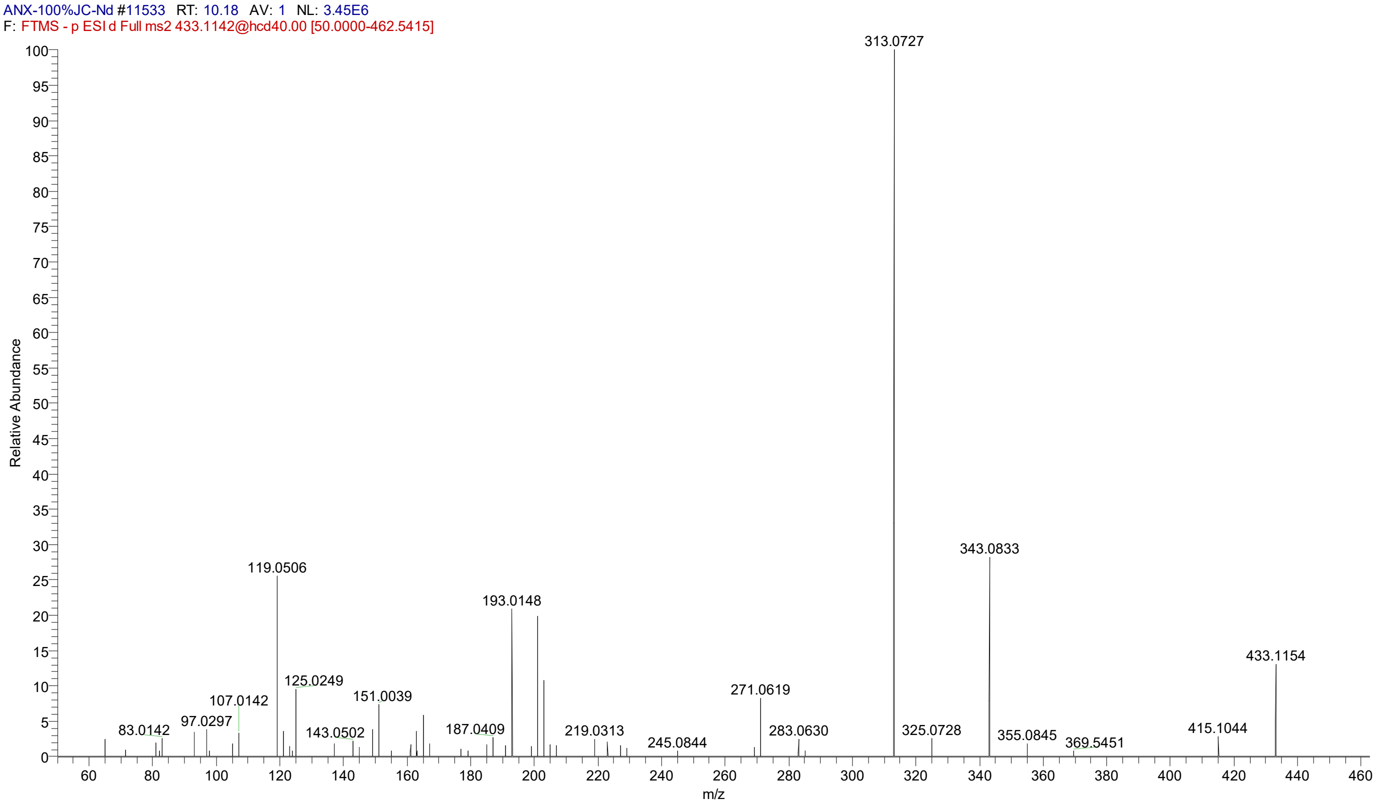

Figure S6. MS² fragmentation spectra (CID) of hemiphloin

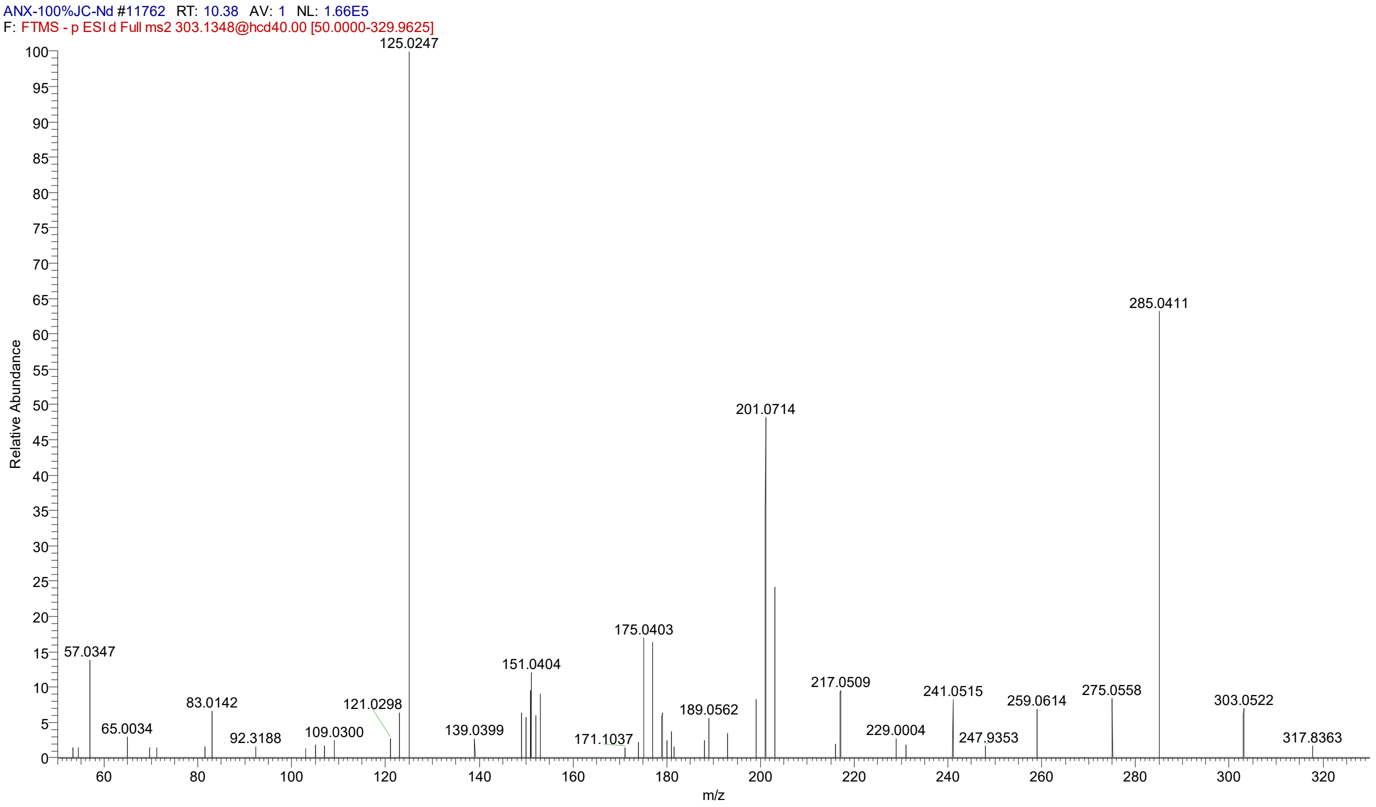

Figure S7. MS² fragmentation spectra (CID) of taxifolin

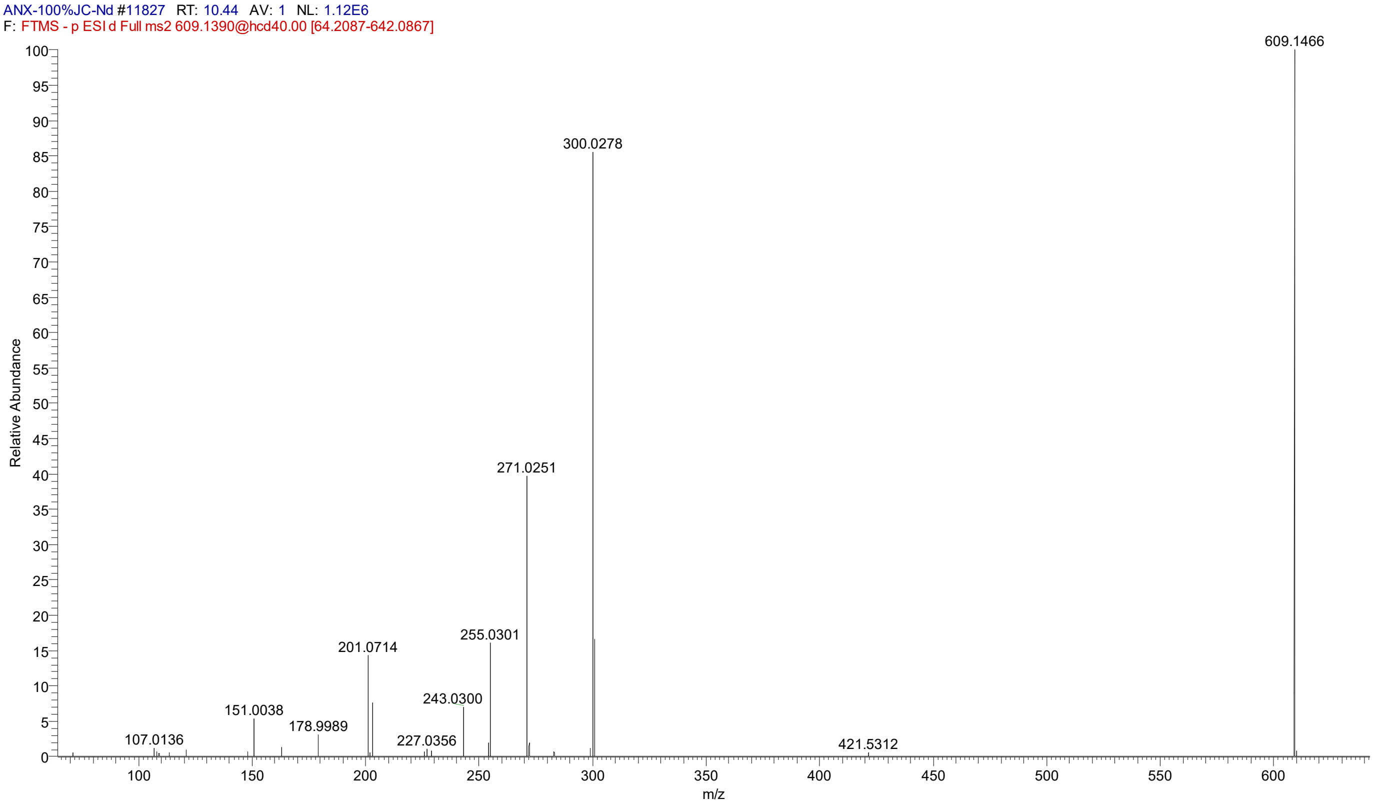

Figure S8. MS² fragmentation spectra (CID) of rutin

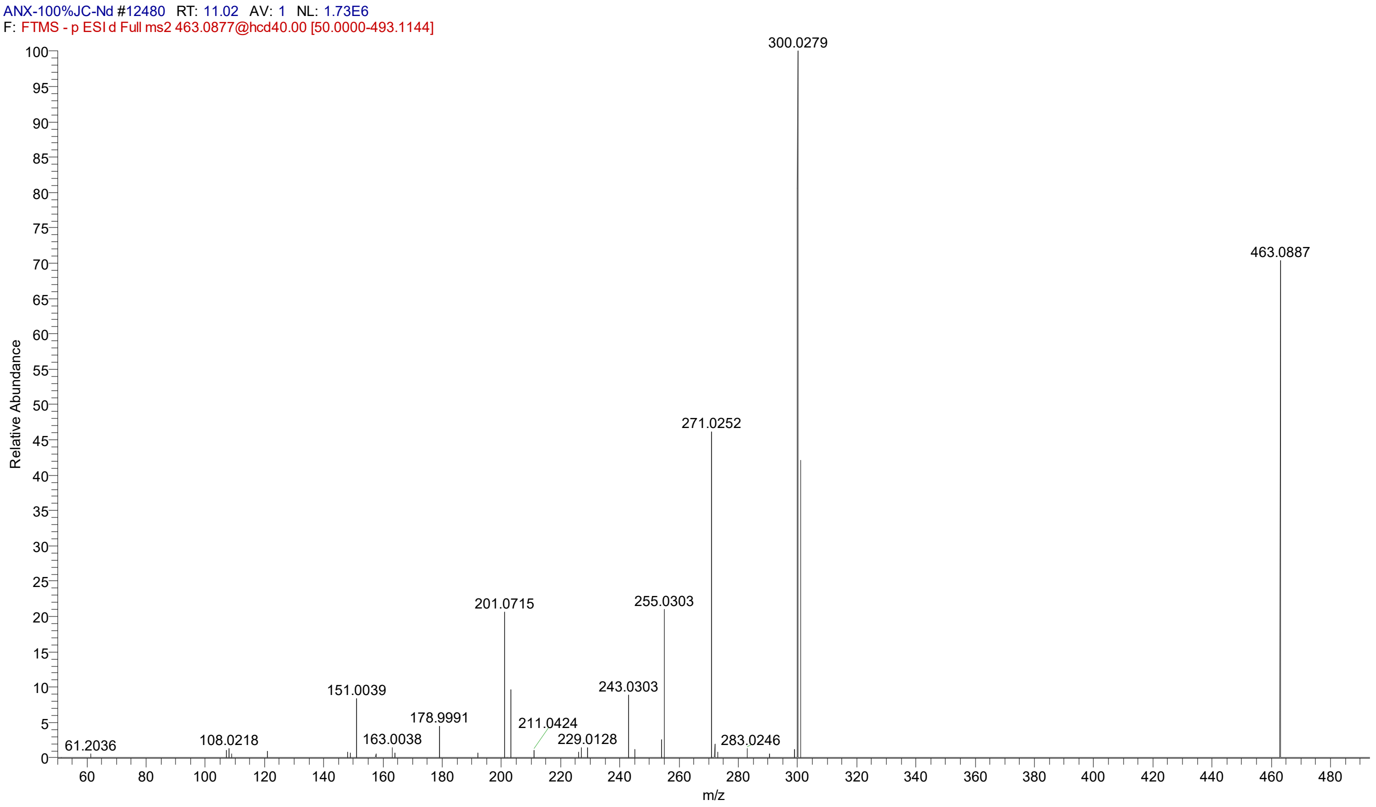

Figure S9. MS^2 fragmentation spectra (CID) of quercetin–3*β*–D–glucoside

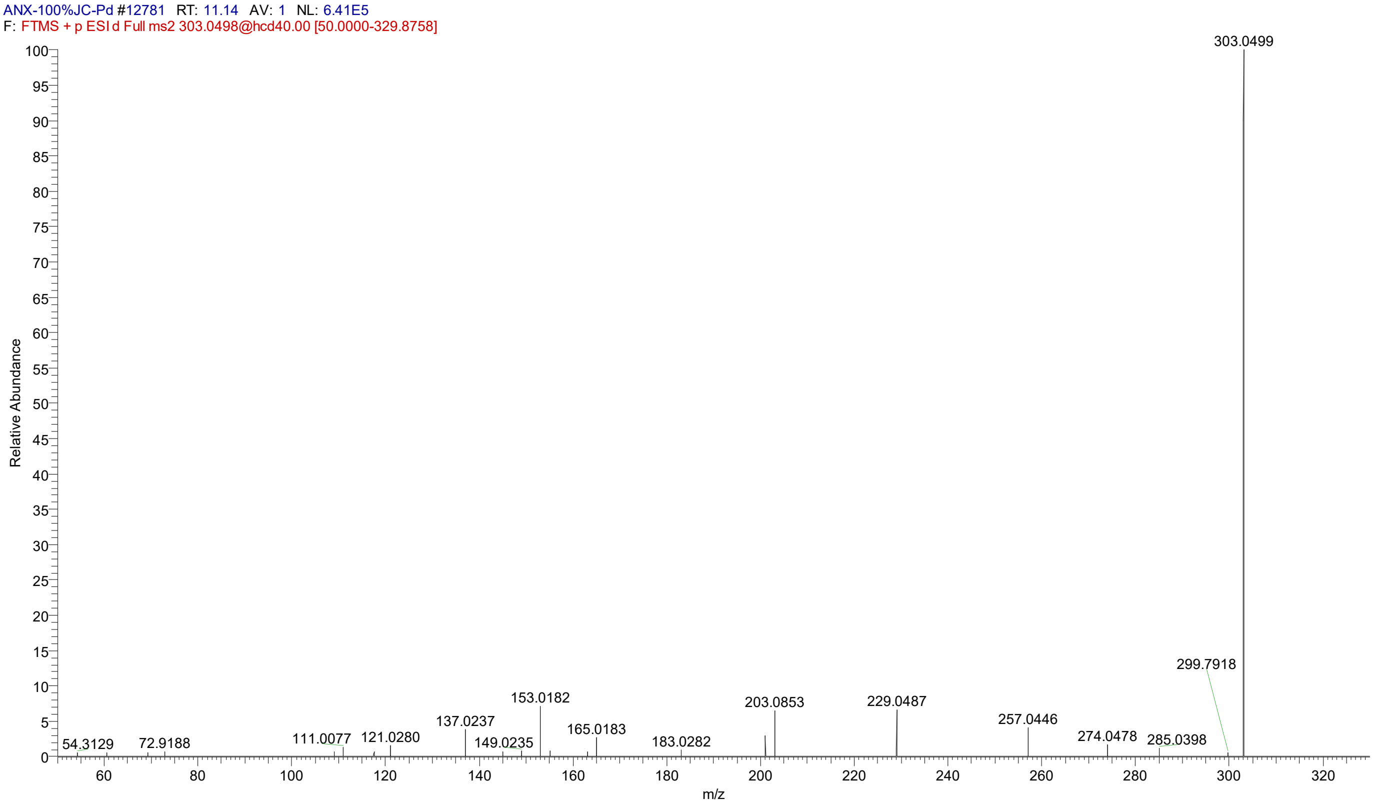

Figure S10. MS^2 fragmentation spectra (CID) of quercetin

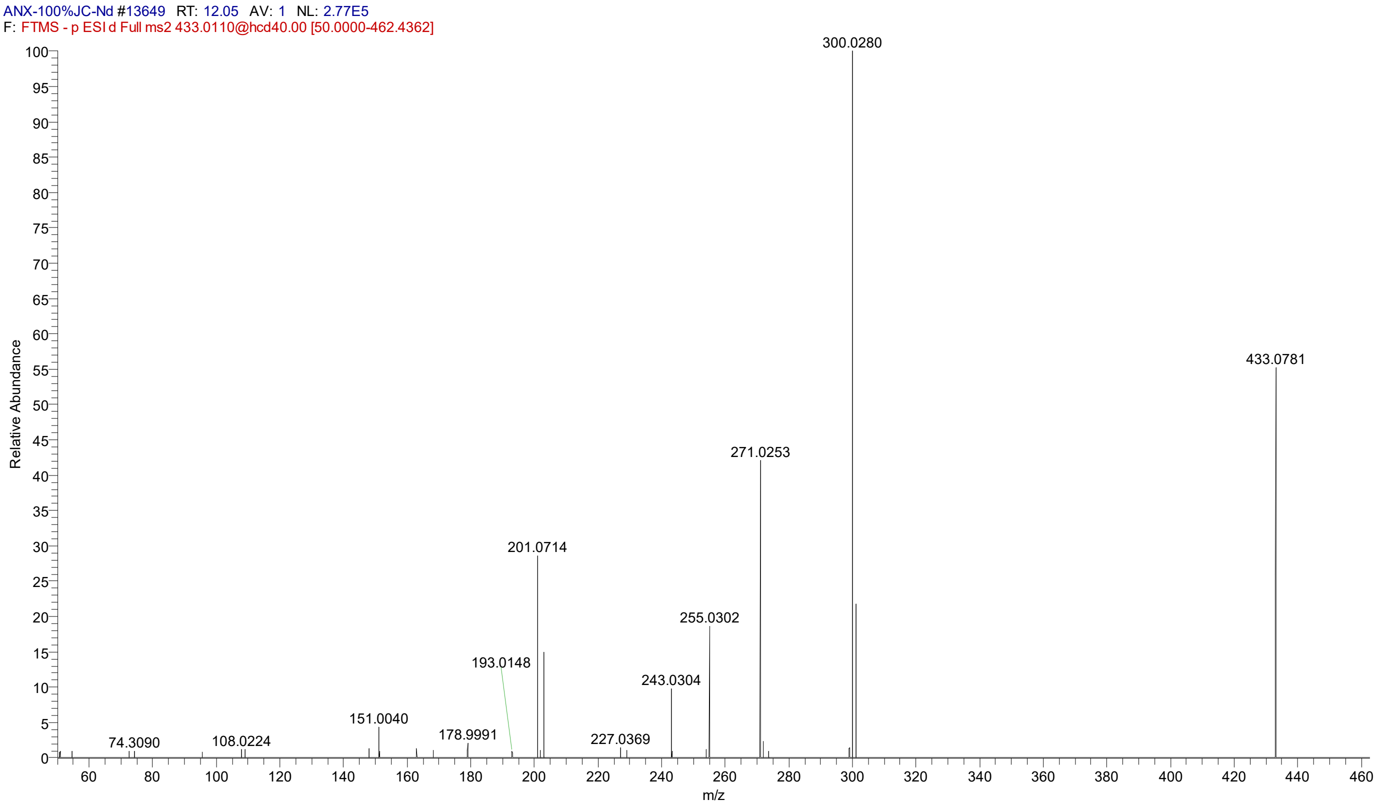

Figure S11. MS² fragmentation spectra (CID) of quercetin–3–Arabinoside

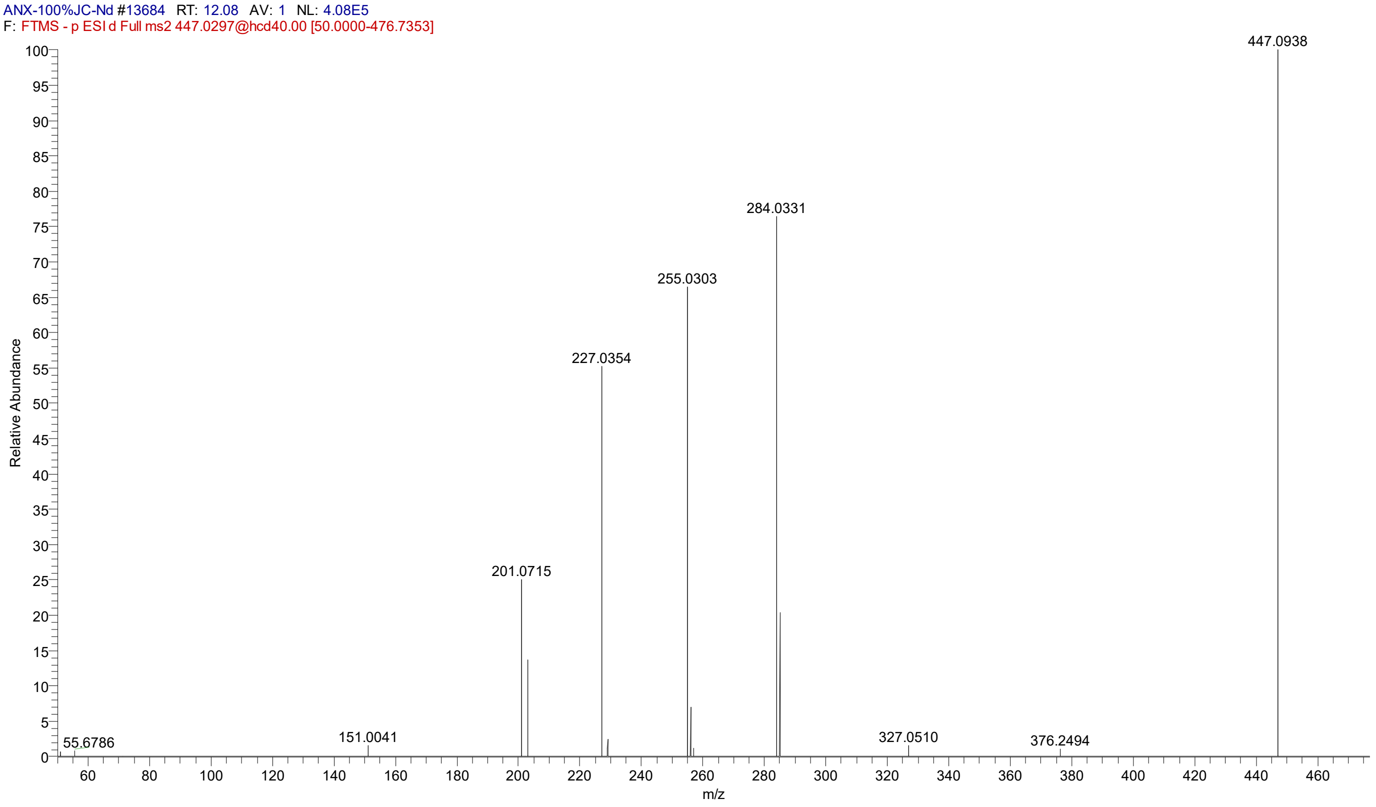

Figure S12. MS² fragmentation spectra (CID) of cynaroside

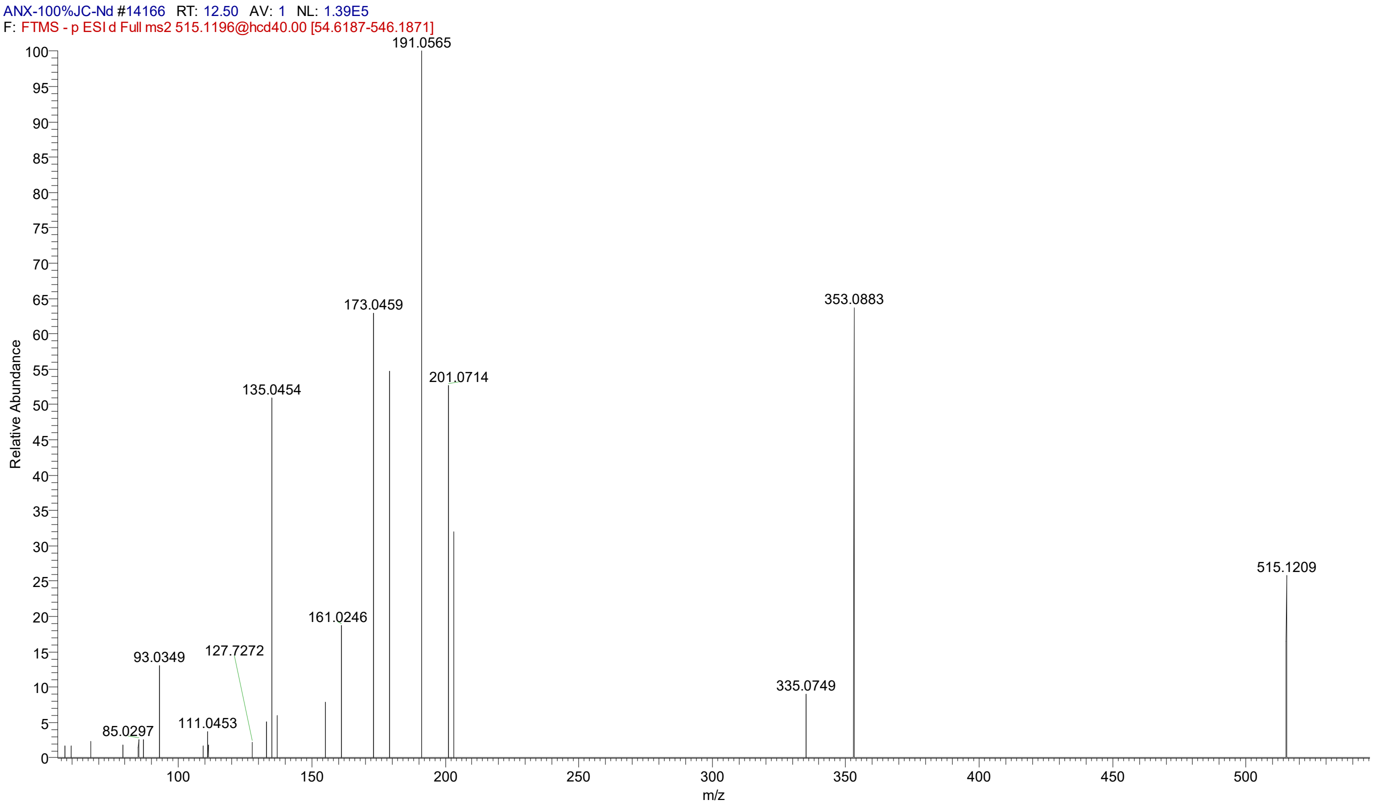

Figure S13. MS² fragmentation spectra (CID) of 1,3–Dicaffeoylquinic acid

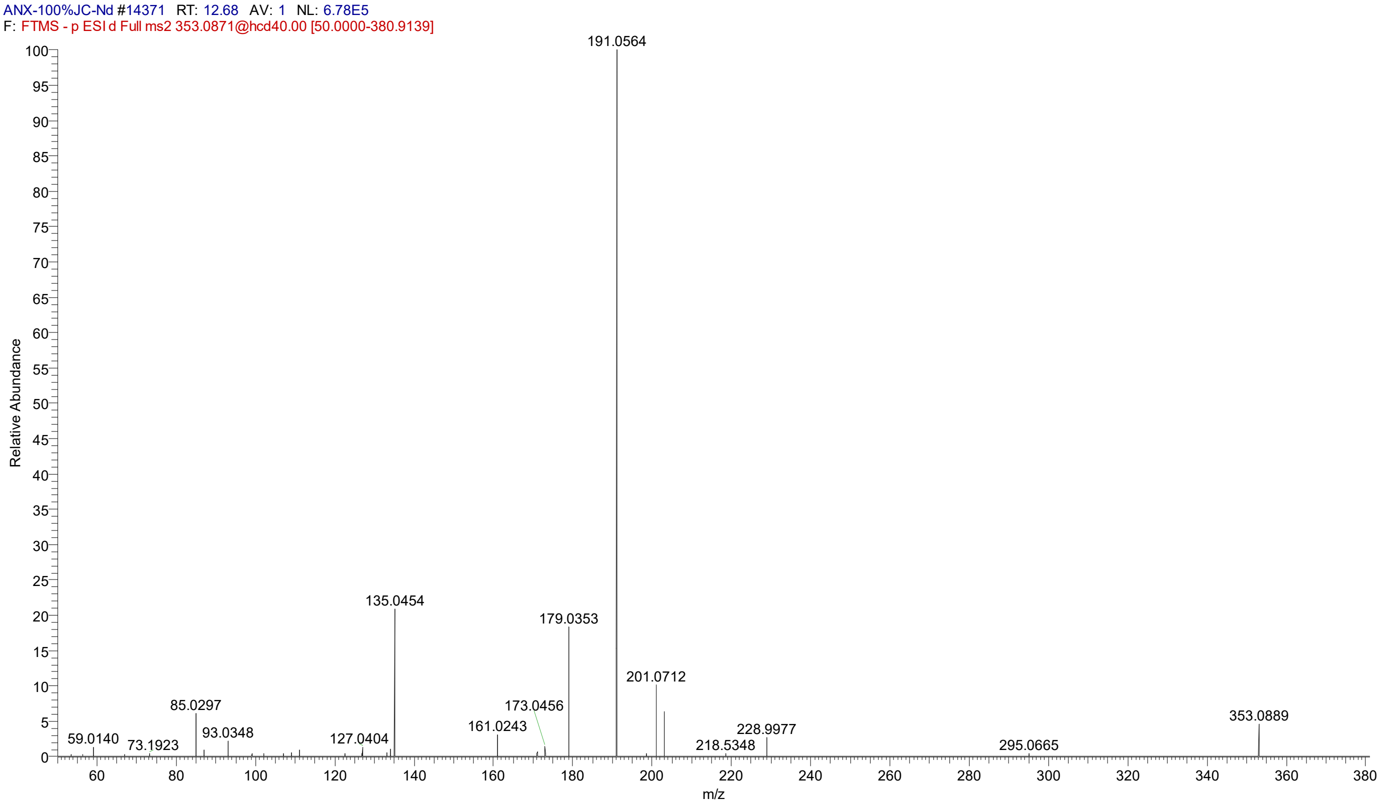

Figure S14. MS² fragmentation spectra (CID) of chlorogenic acid

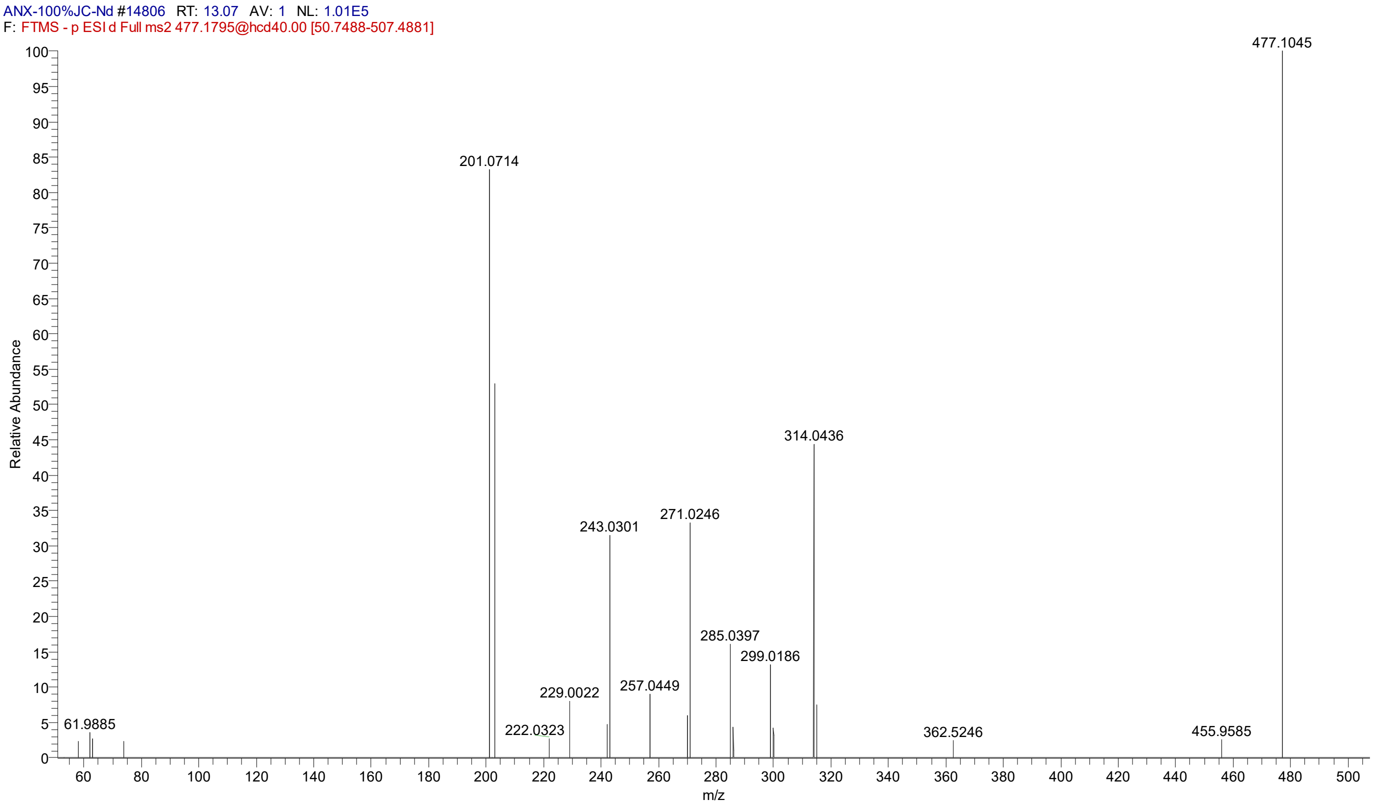

Figure S15. MS² fragmentation spectra (CID) of isorhamnetin–3–O–glucoside

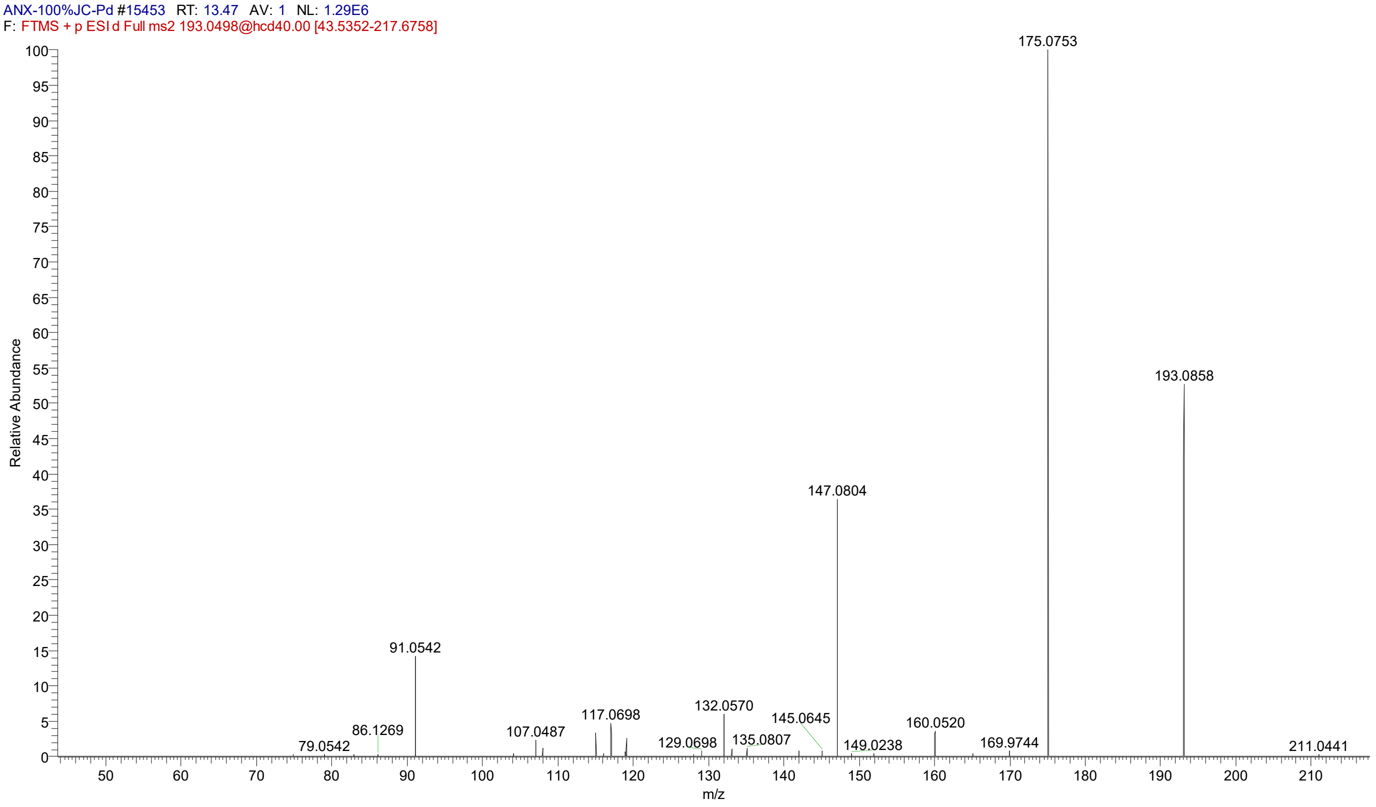

Figure S16. MS² fragmentation spectra (CID) of 4–oxo–5–phenylpentanoic acid

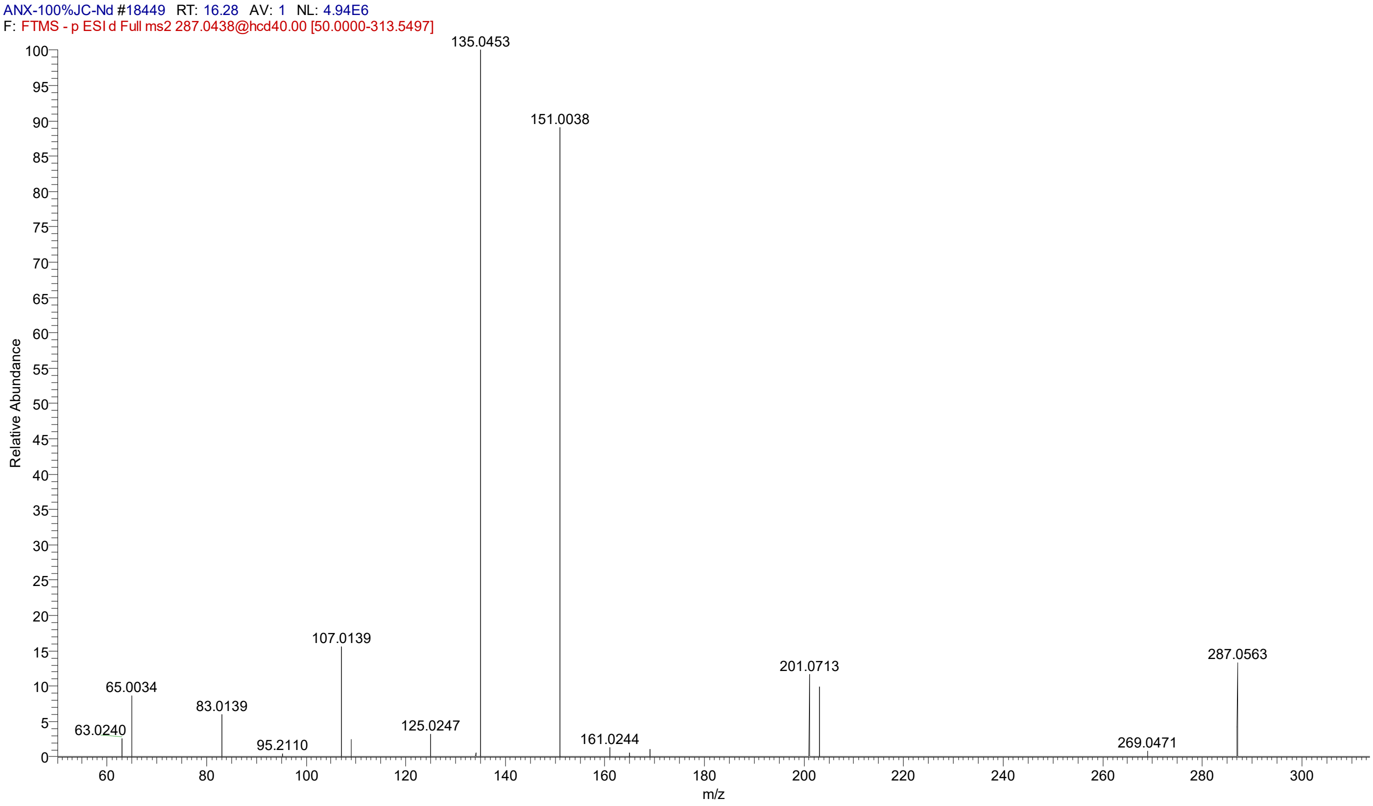

Figure S17. MS² fragmentation spectra (CID) of eriodictyol

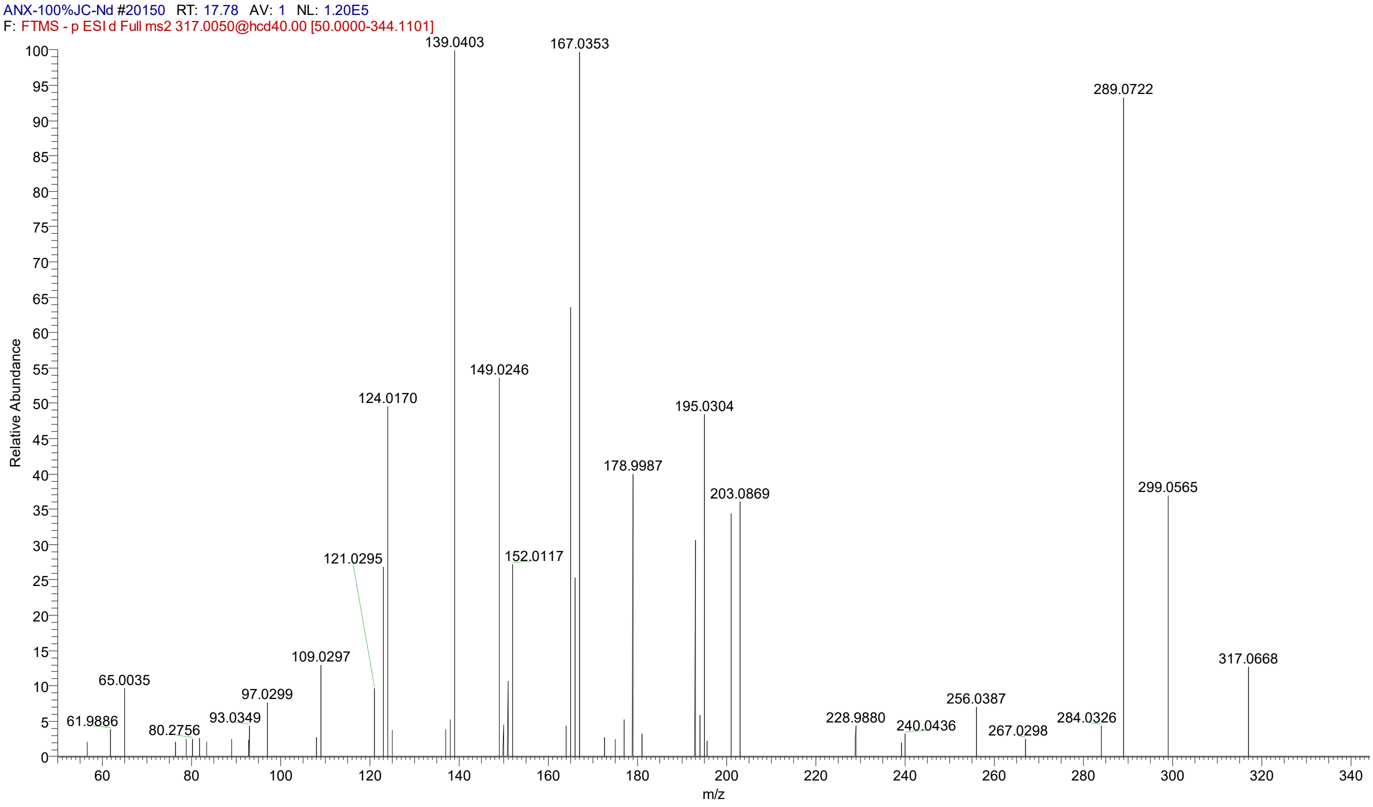

Figure S18. MS² fragmentation spectra (CID) of padmatin

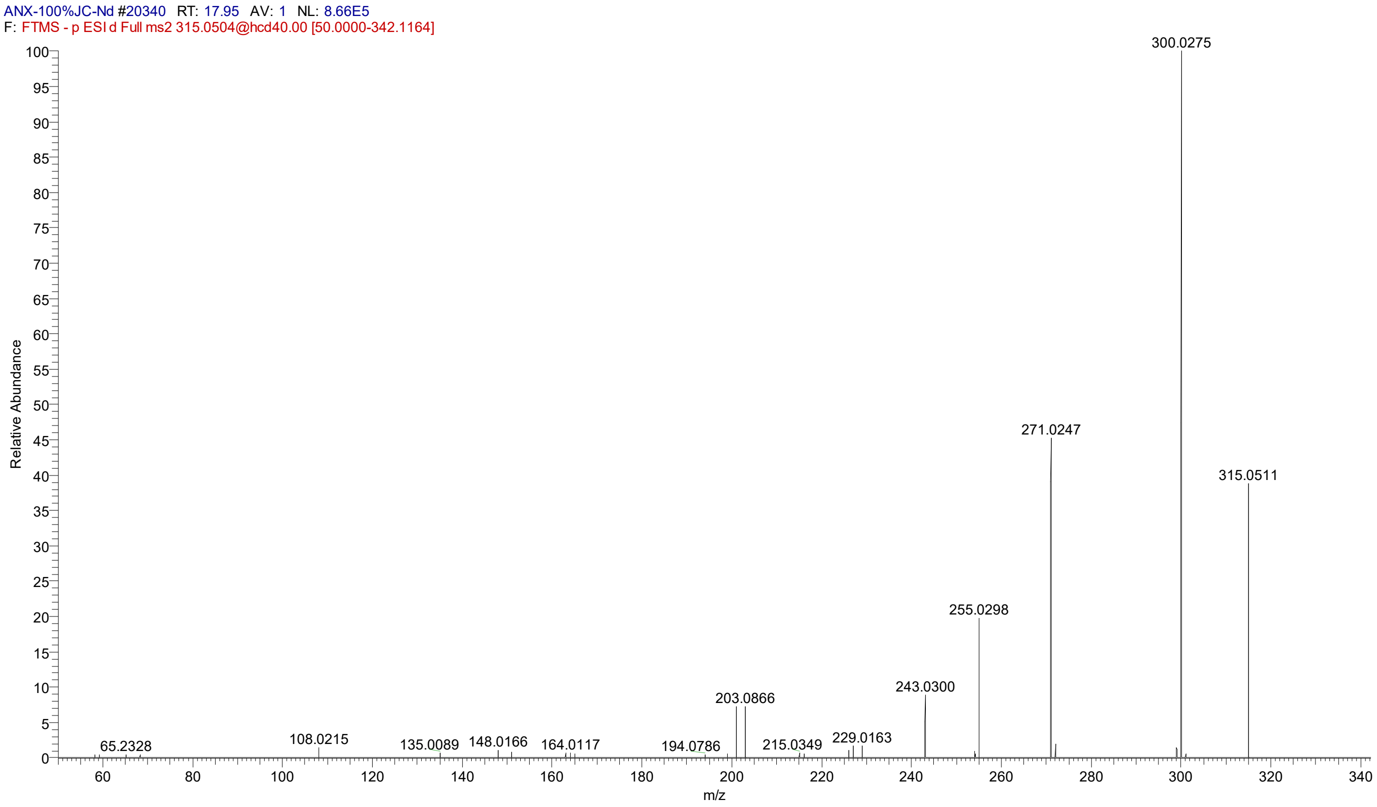

Figure S19. MS² fragmentation spectra (CID) of isorhamnetin

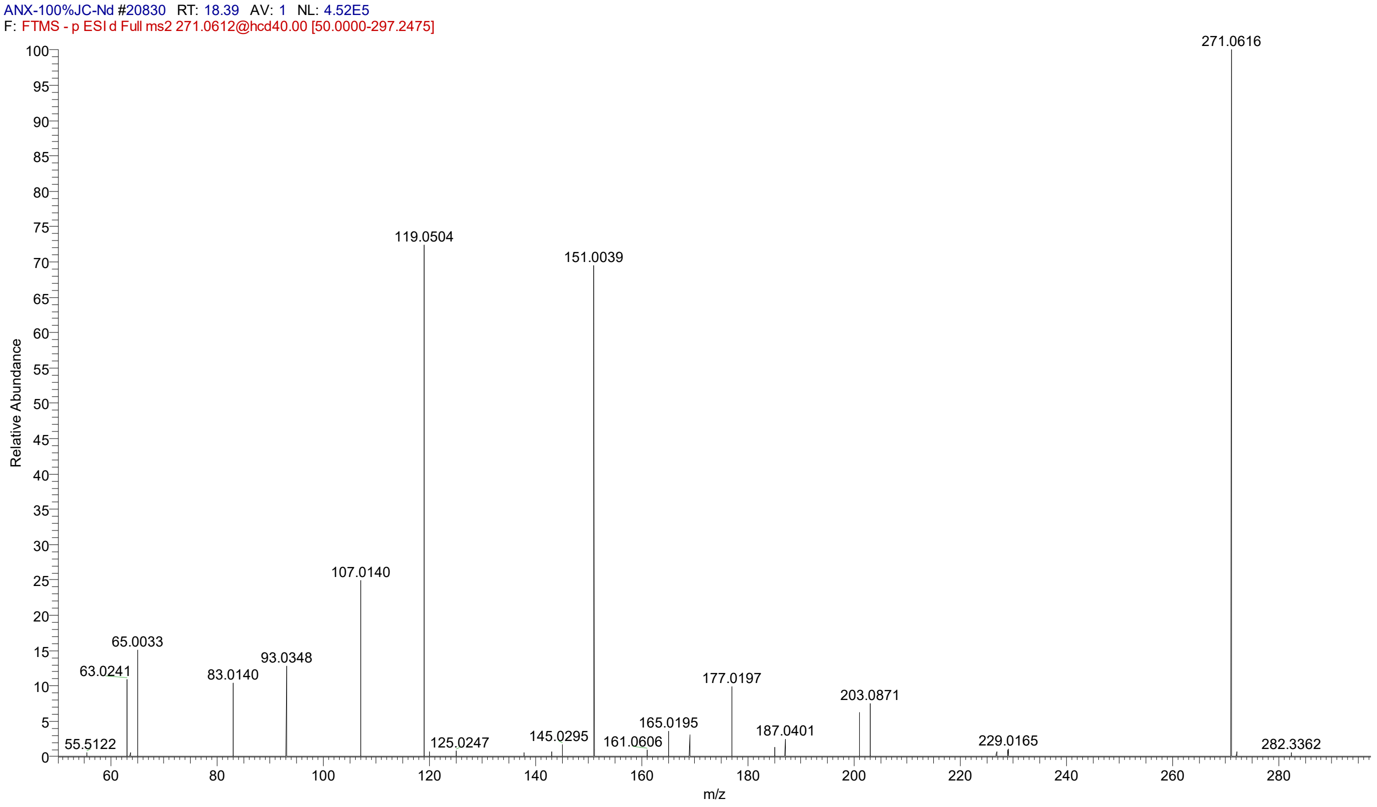

Figure S20. MS² fragmentation spectra (CID) of naringenin

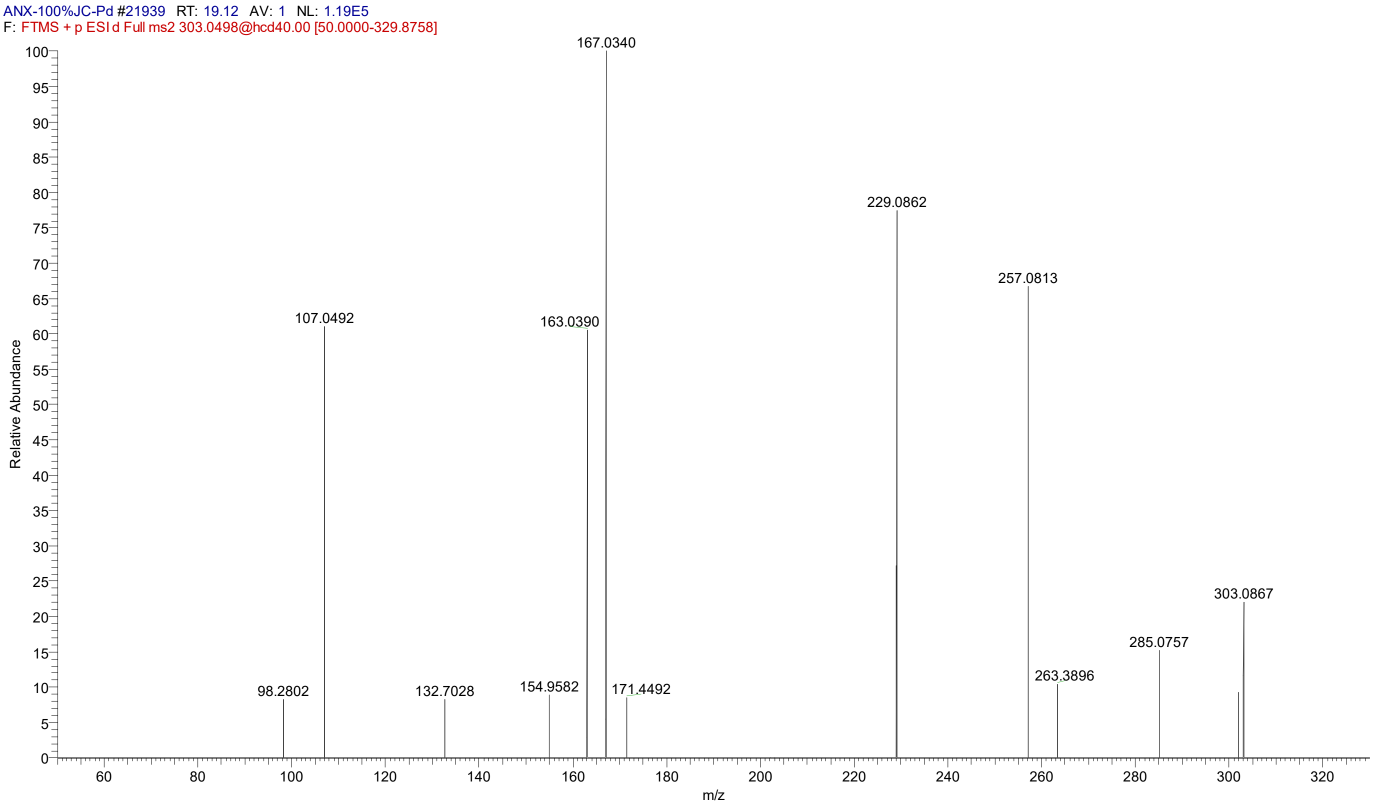

Figure S21. MS² fragmentation spectra (CID) of 7–O–Methylaromadendrin

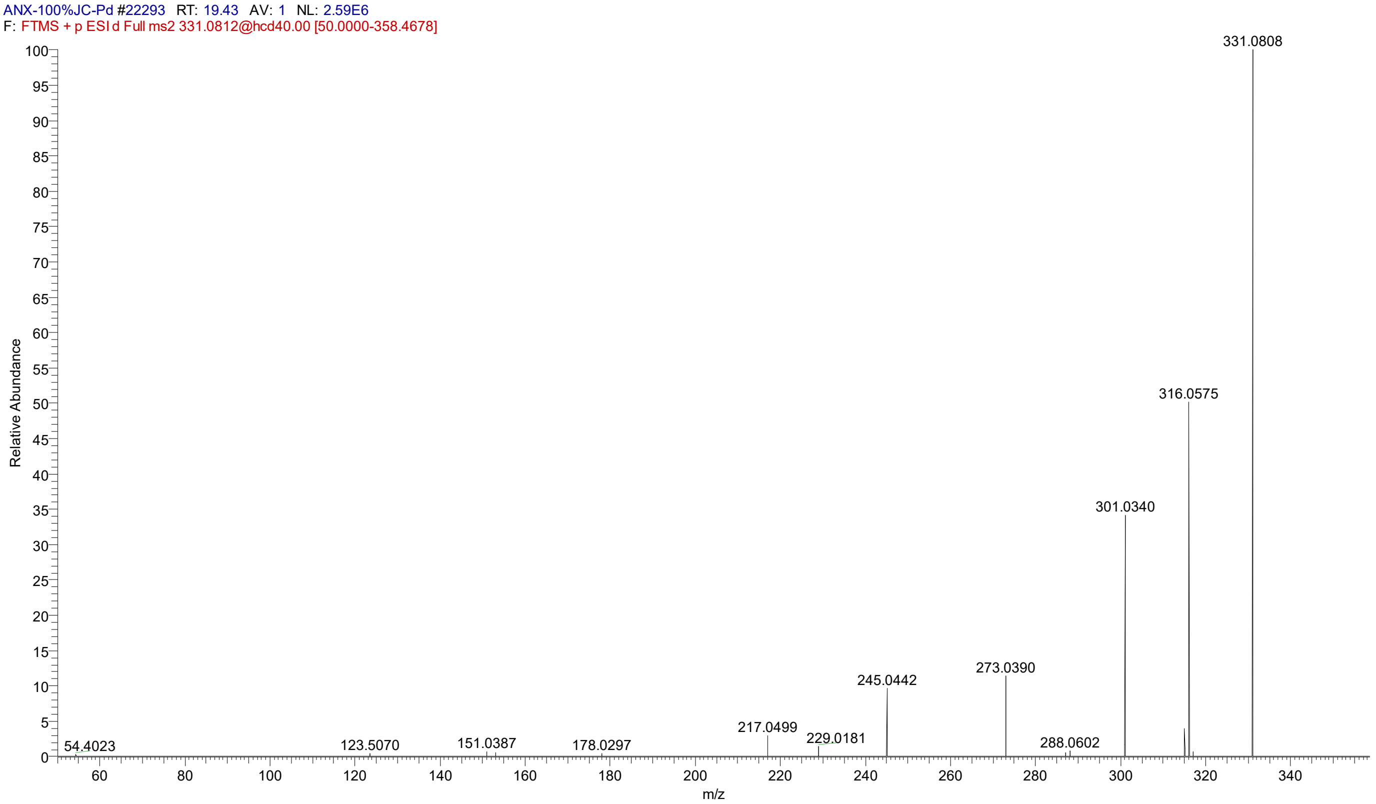

Figure S22. MS² fragmentation spectra (CID) of aurantio–obtusin

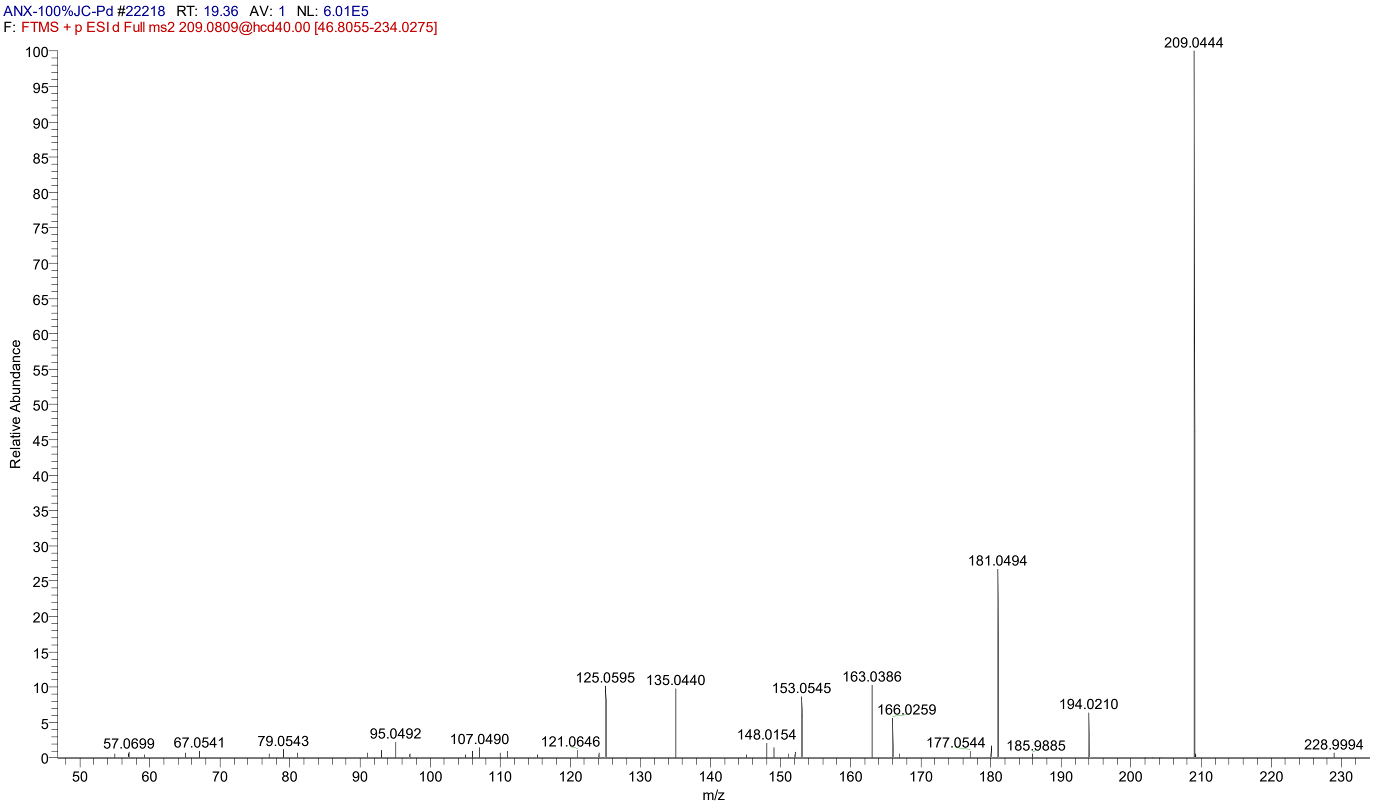

Figure S23. MS² fragmentation spectra (CID) of fraxetin

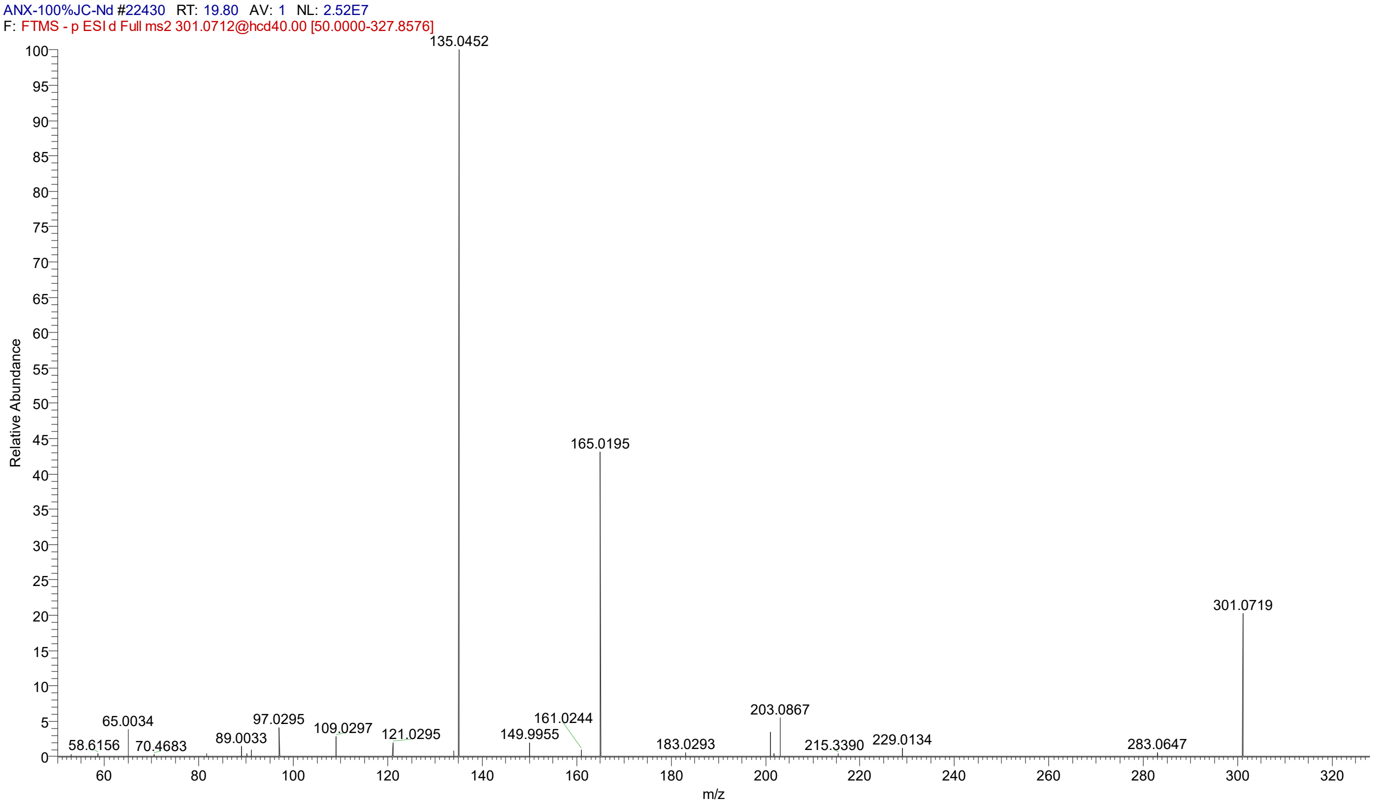

Figure S24. MS² fragmentation spectra (CID) of hematoxylin

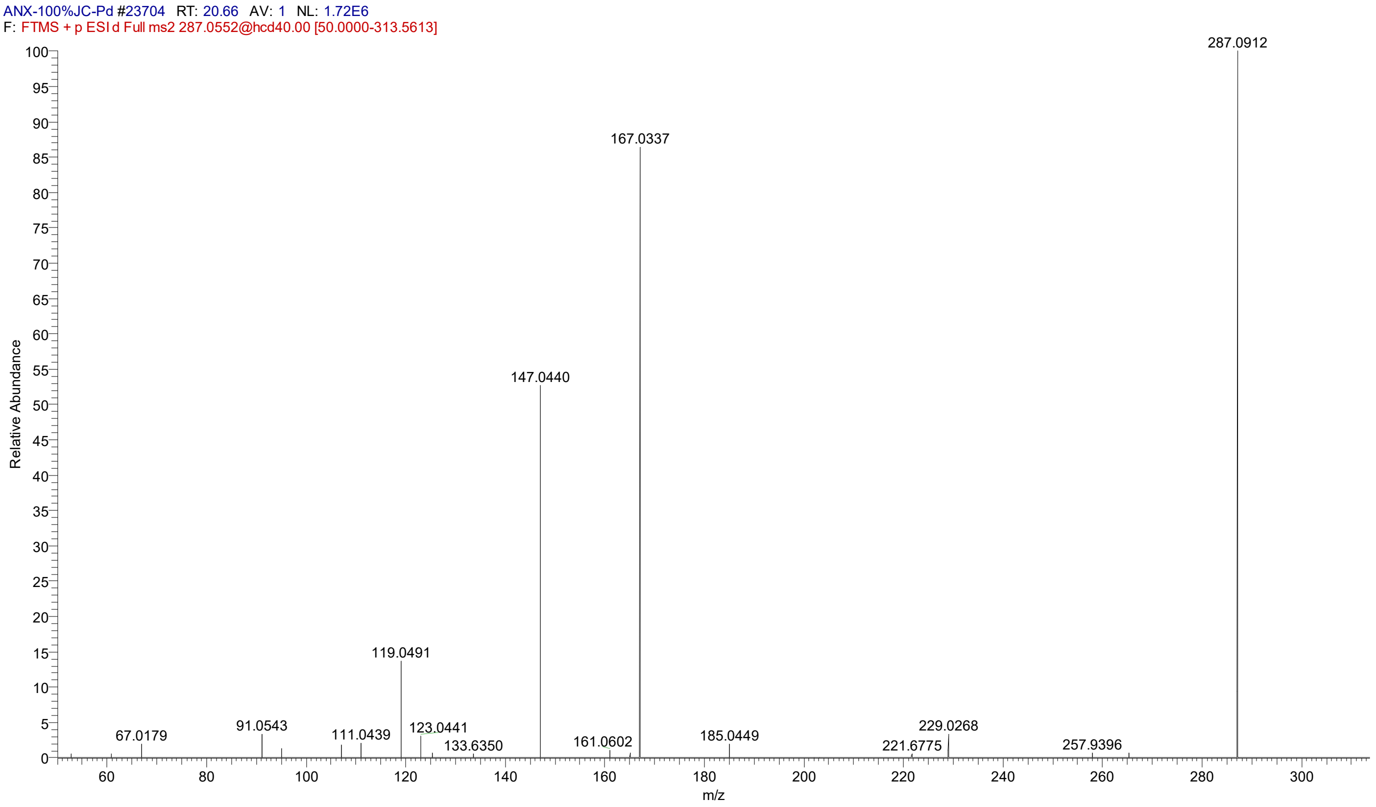

Figure S25. MS² fragmentation spectra (CID) of sakuranetin

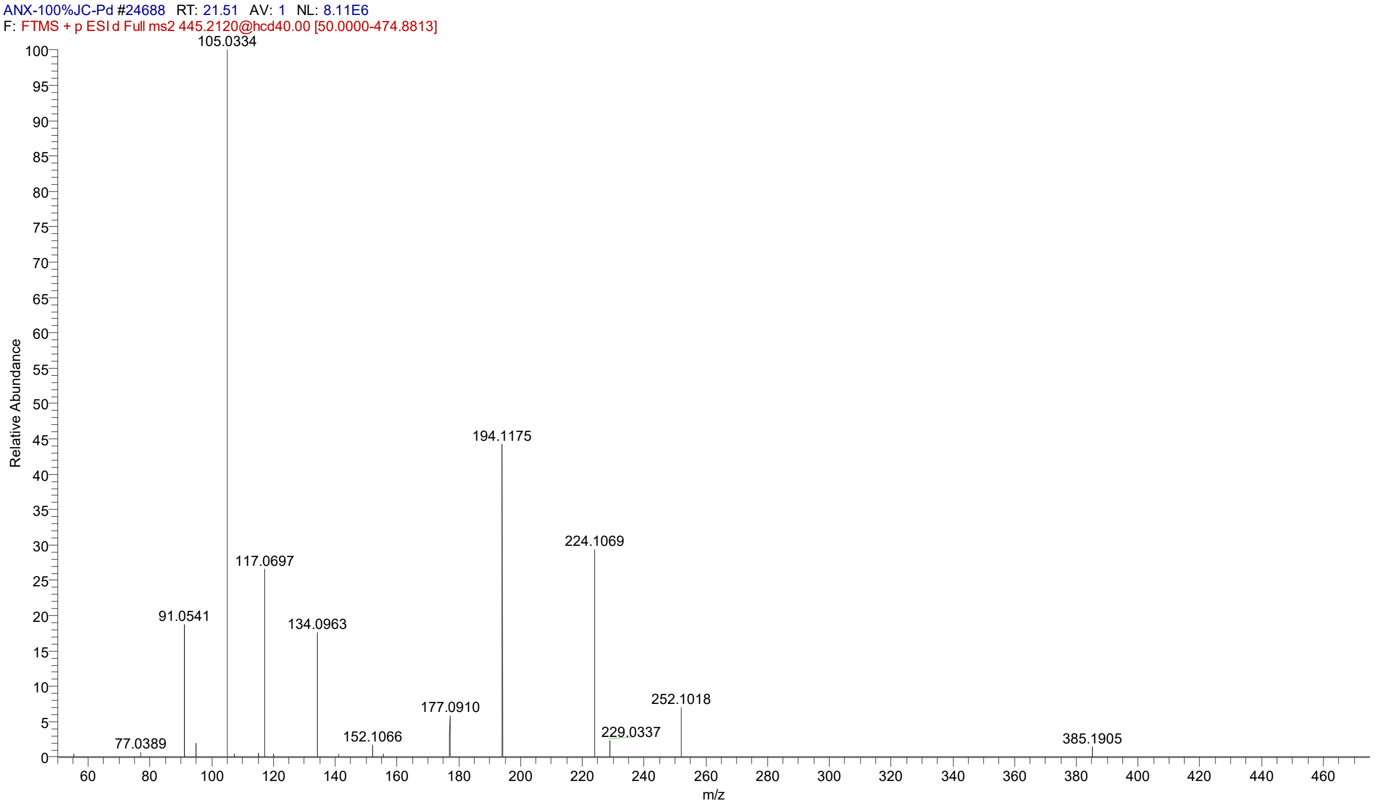

Figure S26. MS² fragmentation spectra (CID) of aurantiamide acetate

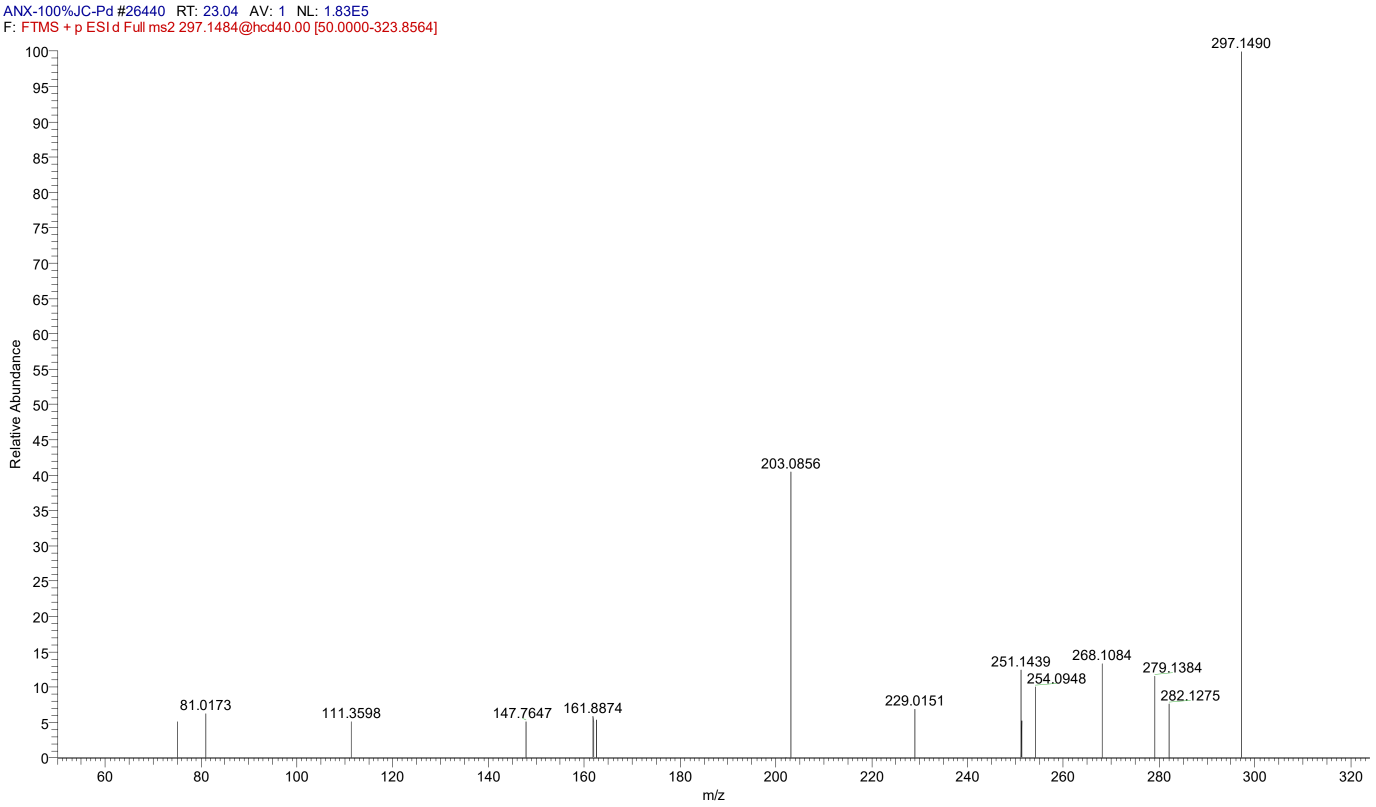

Figure S27. MS^2 fragmentation spectra (CID) of cryptotanshinone

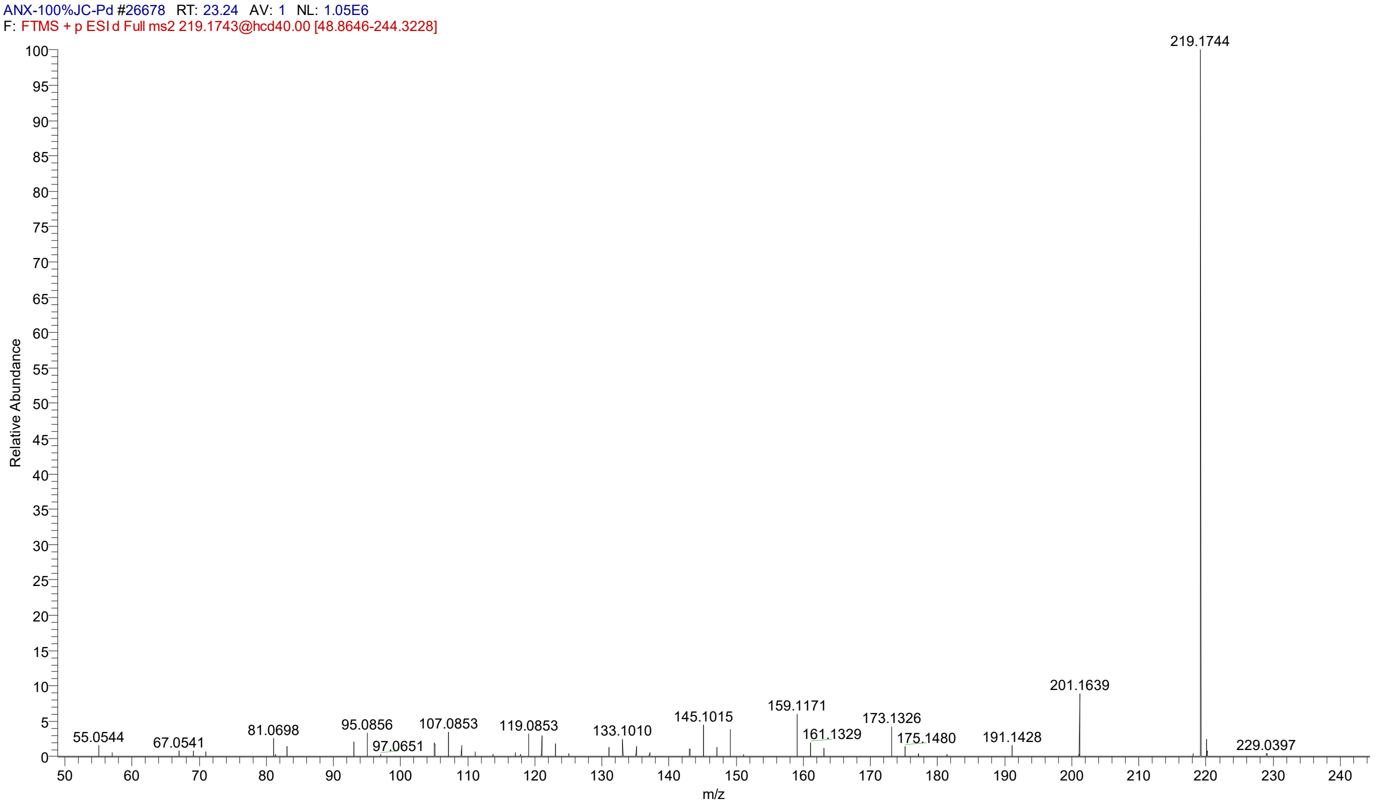

Figure S28. MS^2 fragmentation spectra (CID) of nootkatone

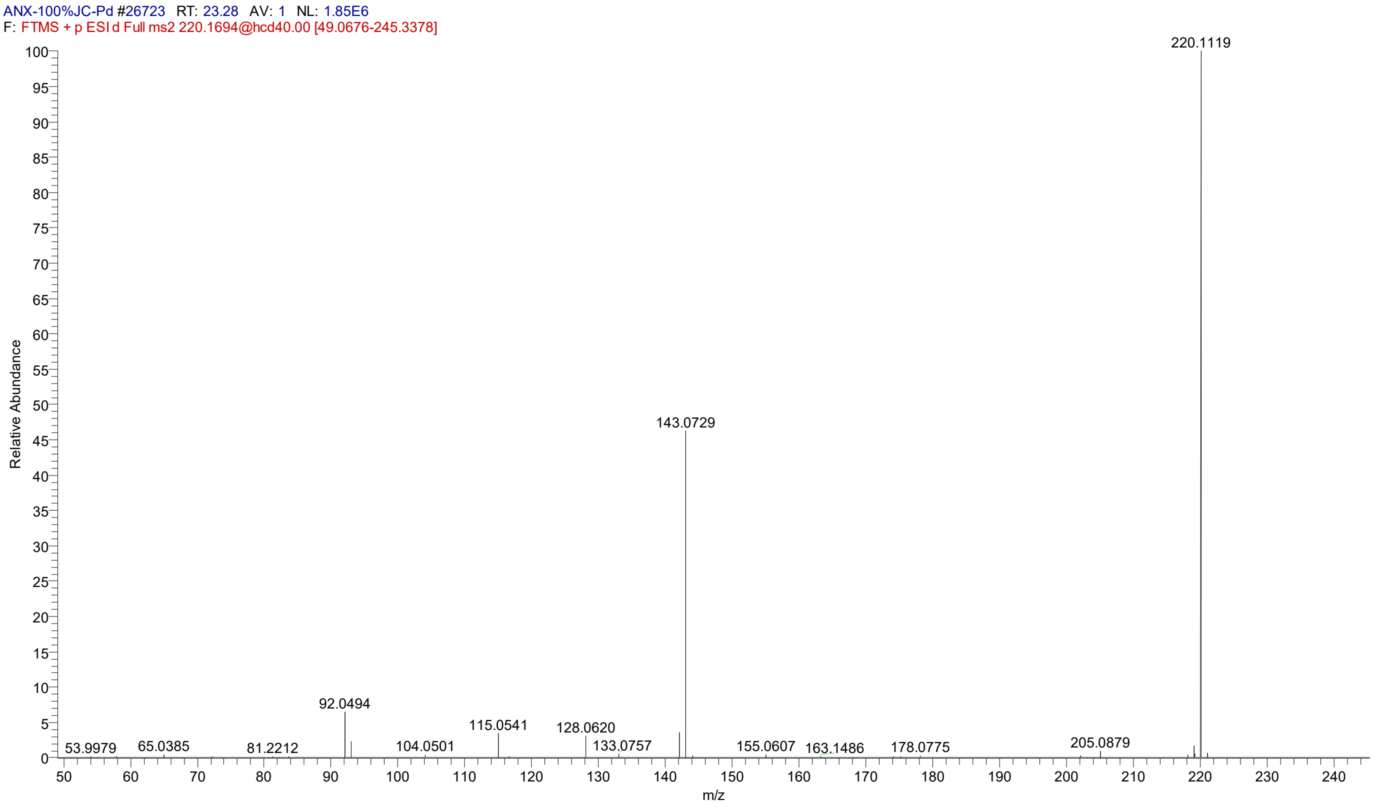

Figure S29. MS² fragmentation spectra (CID) of N–Phenyl–1–naphthylamine

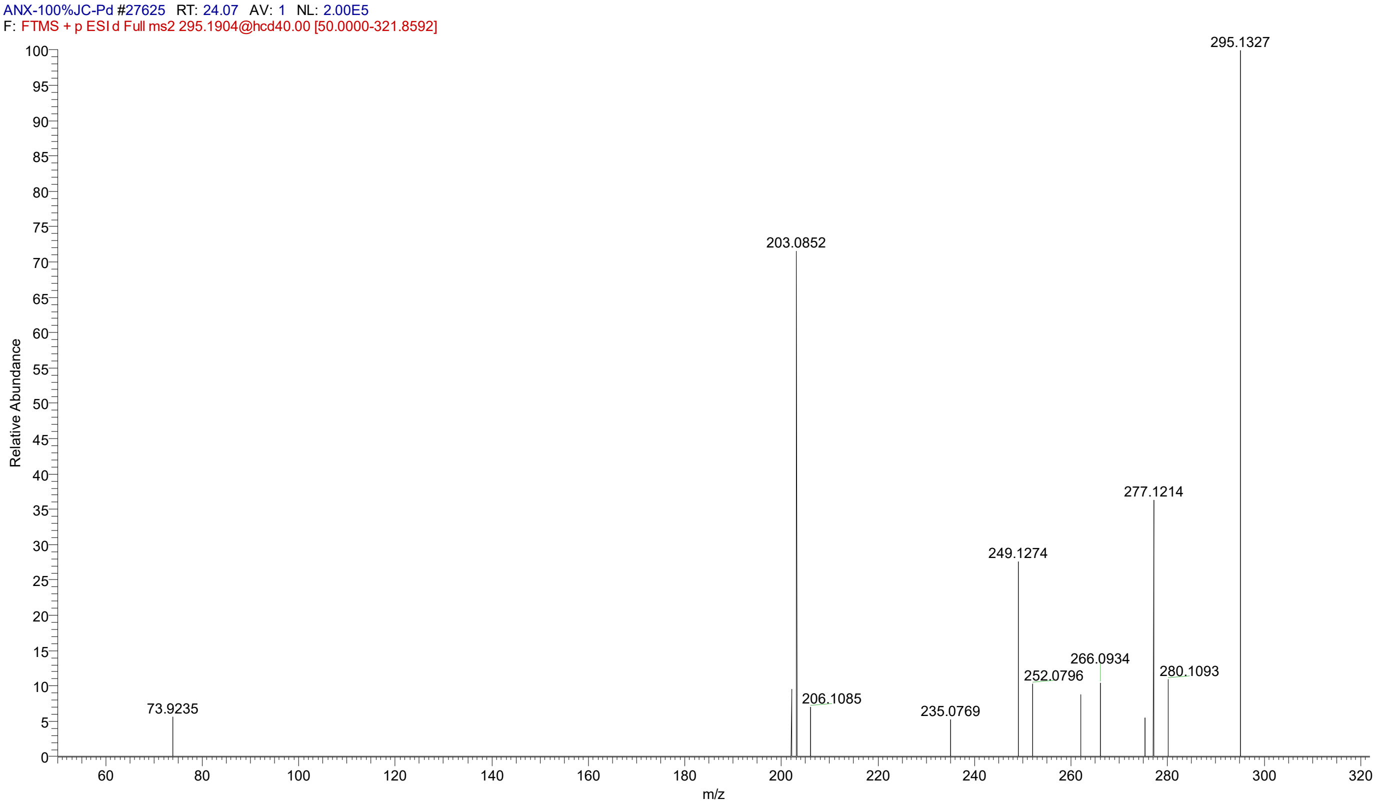

Figure S30. MS² fragmentation spectra (CID) of tanshinone IIA

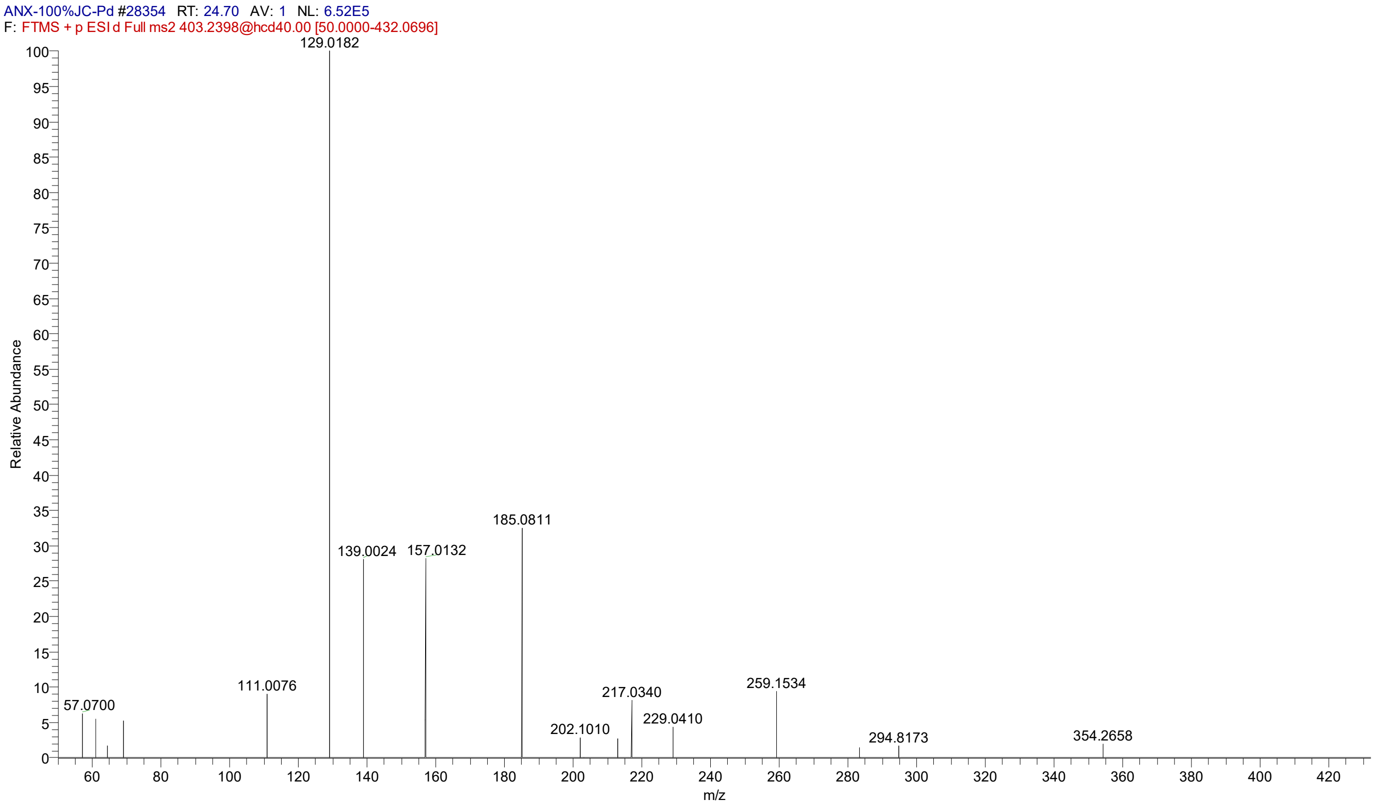

Figure S31. MS^2 fragmentation spectra (CID) of citroflex A–4

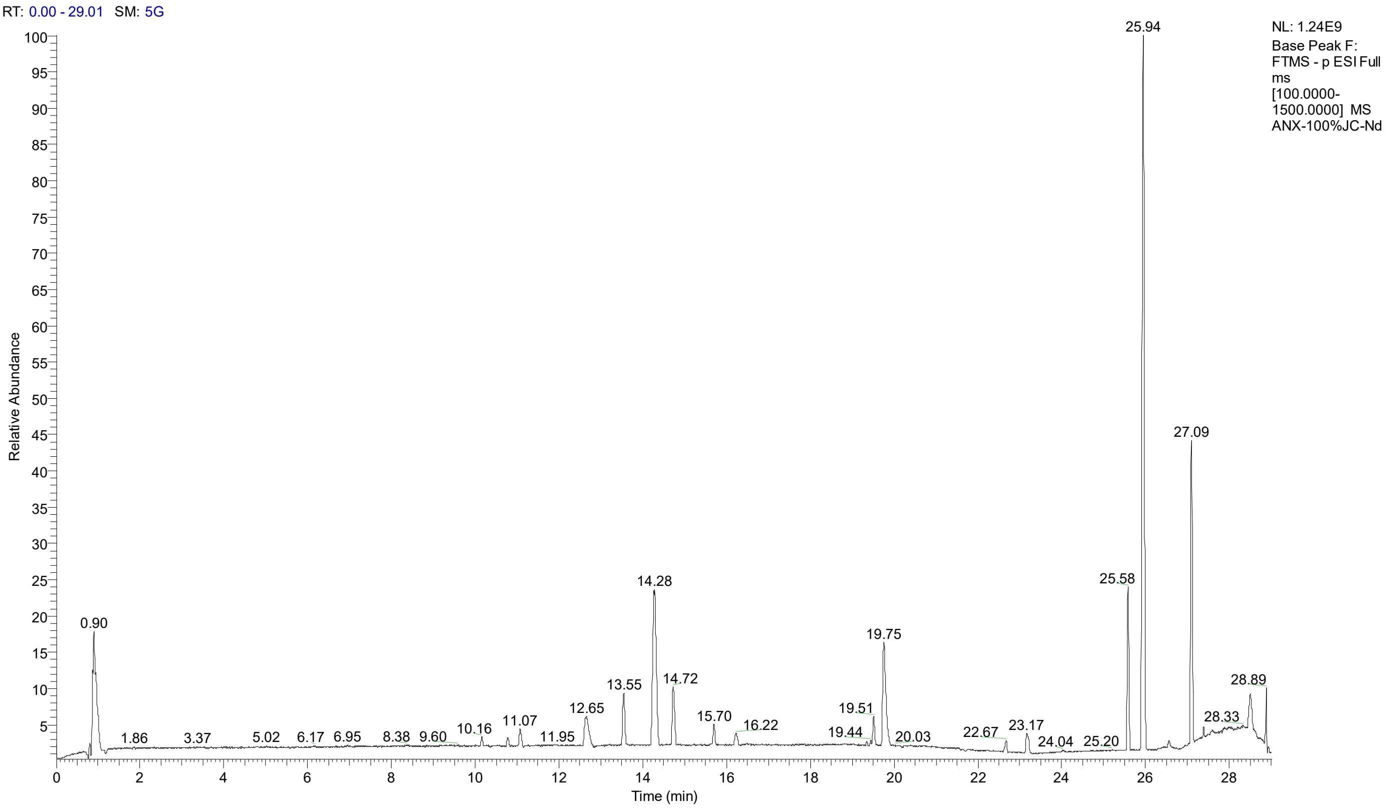

Figure S32. TICs of *Blumea balsamifera* (L.) DC. in negative ion mode

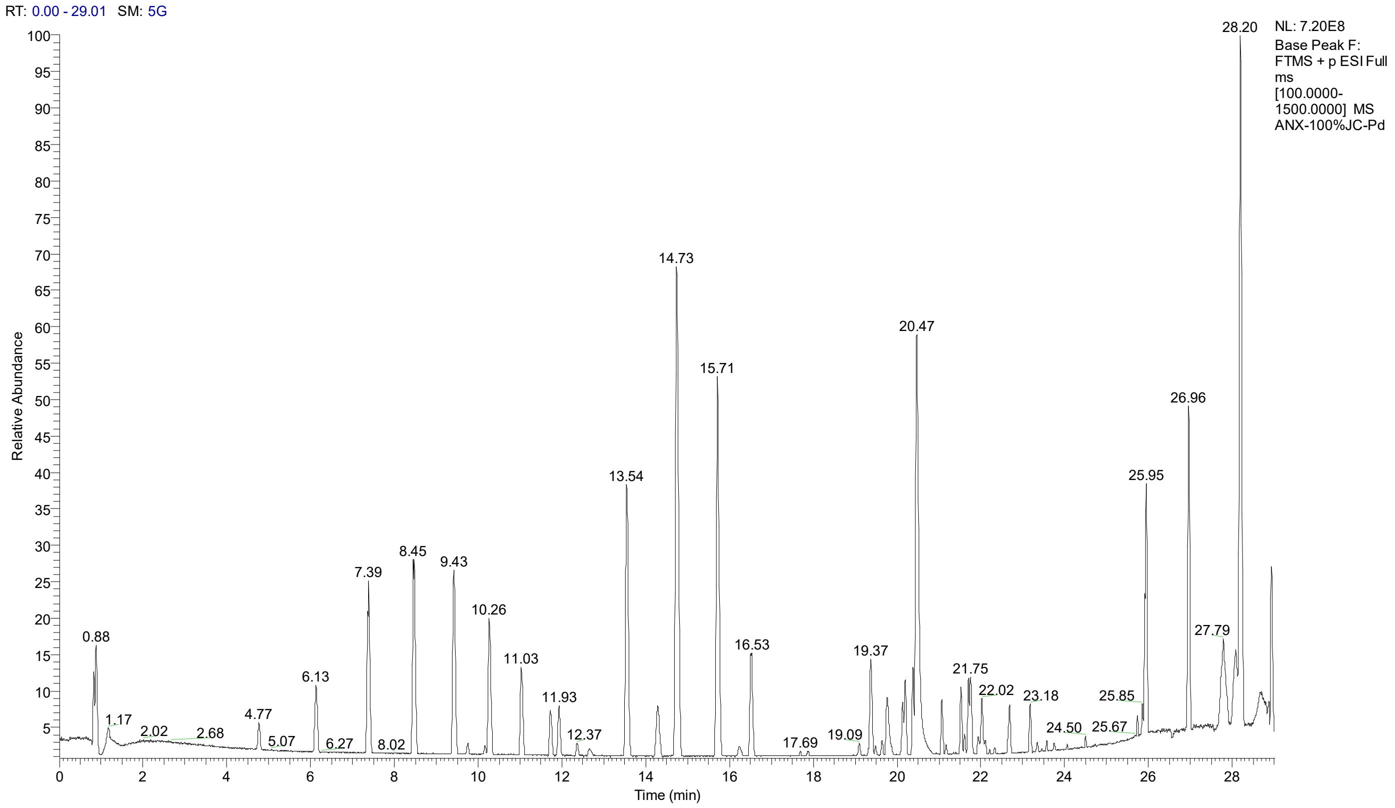

Figure S33. TICs of *Blumea balsamifera* (L.) DC. in positive ion mode
